# The basic helix‐loop‐helix transcription factor, OsPIL15, regulates grain size via directly targeting a purine permease gene *OsPUP7* in rice

**DOI:** 10.1111/pbi.13075

**Published:** 2019-01-24

**Authors:** Xin Ji, Yanxiu Du, Fei Li, Hongzheng Sun, Jing Zhang, Junzhou Li, Ting Peng, Zeyu Xin, Quanzhi Zhao

**Affiliations:** ^1^ Henan Key Laboratory of Rice Biology Collaborative Innovation Center of Henan Grain Crops Henan Agricultural University Zhengzhou China

**Keywords:** rice, *OsPIL15*, grain size, *OsPUP7*, cytokinin

## Abstract

As members of the basic helix‐loop‐helix transcription factor families, phytochrome‐interacting factors regulate an array of developmental responses ranging from seed germination to plant growth. However, little is known about their roles in modulating grain development. Here, we firstly analyzed the expression pattern of rice *OsPIL* genes in grains and found that *OsPIL15* may play an important role in grain development. We then generated knockout (KO) *OsPIL15* lines in rice using CRISPR/Cas9 technology, the silencing expression of *OsPIL15* led to increased numbers of cells, which thus enhanced grain size and weight. Moreover, overexpression and suppression of *OsPIL15* in the rice endosperm resulted in brown rice showing grain sizes and weights that were decreased and increased respectively. Further studies indicated that OsPIL15 binds to N1‐box (CACGCG) motifs of the purine permease gene *OsPUP7* promoter. Measurement of isopentenyl adenosine, a bioactive form of cytokinin (CTK), revealed increased contents in the *OsPIL15*‐KO spikelets compared with the wild‐type. Overall, our results demonstrate a possible pathway whereby OsPIL15 directly targets *OsPUP7*, affecting CTK transport and thereby influencing cell division and subsequent grain size. These findings provide a valuable insight into the molecular functions of *OsPIL15* in rice grains, highlighting a useful genetic improvement leading to increased rice yield.

## Introduction

Rice (*Oryza sativa* L.) is one of the most important food crops worldwide. Grain size is an important and complex agronomic trait that determines yield potential and is controlled by polygenes. A number of major quantitative trait loci (QTLs) controlling grain size have been successfully isolated and characterized, such as *GS3* (Fan *et al*., 2006; Mao *et al*., 2010), *GS5* (Li *et al*., 2011), *GS9* (Zhao *et al*., 2018), *GW2* (Song *et al*., 2007), *GW5* (Shomura *et al*., 2008; Weng *et al*., 2008), *GW7* (Wang *et al*., 2015), *GW8* (Wang *et al*., 2012) and *TGW6* (Ishimaru *et al*., 2013). Furthermore, recent studies have revealed that a number of transcription factors act as key grain size regulators. For example, *GLW7* encodes the transcription factor, OsSPL13, which improves grain size by increasing cell expansion in spikelet hulls (Si *et al*., 2016). *GW8* encodes the transcription factor, OsSPL16, which increases grain width and yield by promoting cell division and grain filling (Wang *et al*., 2012). Similarly, *GS2* encodes the transcriptional regulator OsGRF4, regulating grain size by increasing cell expansion and cell proliferation in spikelet hulls (Hu *et al*., 2015). Meanwhile, *qLGY3* encodes the MADS‐domain transcription factor OsMADS1, which is associated with long and slender grains resulting from increased cell division in a longitudinal direction in the outer epidermis (Liu *et al*., 2018). *GL4* encodes a MYB‐like transcription factor and controls grain length by regulating longitudinal cell elongation in the outer and inner glumes in African rice (Wu *et al*., 2017). Moreover, the OsGBP1 transcription factor represses grain length while OsGBP3 enhances grain length (Gong *et al*., 2018). Besides the restriction by the spikelet hull, endosperm growth or grain filling is also crucial to decide the grain size. The transcription factor, *MADS29*, regulates the degradation of maternal tissues and hence grain filling (Yin and Xue, 2012). Knockdown of the transcription factor *OsNF‐YB1*'s expression significantly retards grain filling, leading to small grains (Xu *et al*., 2016). Although a set of transcription factors controlling rice grain size have been identified and characterized, the underlying molecular mechanisms remain to be elucidated.

Basic helix‐loop‐helix (bHLH) transcription factors that contain adjacent basic and HLH regions constitute a large protein family in plants. The basic region is located at the N terminus of the bHLH domain and functions as a DNA‐binding motif. The majority of bHLHs bind to a *cis*‐element known as an E‐box (CANNTG; Pires and Dolan, 2010; Carretero‐Paulet *et al*., 2010), which plays critical roles in plant development and environmental responses. Recently, a triantagonistic bHLH system has been reported to regulate cell elongation in *Arabidopsis* (Ikeda *et al*., 2012). Maize PIF transcription factor *ZmPIF1* causes an increase in grain yield via increases in tiller and panicle numbers in transgenic rice (Gao *et al*., 2018). In rice, the bHLH transcription factor APG (also known as *OsPIL16*) functions antagonistically with two atypical bHLH transcription factors, PGL1 and PGL2, to influence grain length (Heang and Sassa, 2012a,2012b). Overexpression of *OsbHLH107* enhances grain size by influencing cell number in a longitudinal direction in the spikelet hulls (Yang *et al*., 2018). A novel protein complex consisting of OsBUL1, LO9‐177 and OsBC1 is associated with the HLH‐bHLH system, having a positive role in grain size through cell elongation in rice (Jang *et al*., 2017). As a member of the *bHLH* gene family in rice, *An‐1* regulates awn development, cell division and grain size (Luo *et al*., 2013). Hence, the bHLH transcription factors also have a significant role in regulating grain size in rice.

Phytochrome‐interacting factors (PIFs) are encoded by a subset of bHLH transcription factors and regulate the expression of their target genes by interacting with G‐box or PIF‐binding E (PBE) box motifs in their promoter regions (Hornitschek *et al*., 2012; Oh *et al*., 2012; Zhang *et al*., 2013). PIFs have been shown to regulate a number of pathways, including photomorphogenesis, circadian clock, hormone signaling, and biotic and abiotic responses (Paik *et al*., 2017; Quint *et al*., 2016; Shor *et al*., 2017). Among them, PIFs are emerging as integrators of signals from various hormone pathways during plant growth and development. As a member of phytohormones, cytokinin (CTK) also plays an important role in seed development (Jameson and Song, 2016). For example, grain filling of inferior spikelets under postanthesis wetting and severe soil drying was improved via elevated CTK levels in the rice shoot (Zhang *et al*., 2010). Reduced expression of *OsCKX2* causes CTK accumulation in inflorescence meristems, increasing the yield of rice (Ashikari *et al*., 2005), and the disruption of CKX genes in *Arabidopsis* leads to increased seed yield (Bartrina *et al*., 2011). Furthermore, recent study has revealed that PIL5 binds to the upstream regions of the genes encoding the CTK‐responsive transcription factors CRF1, CRF2 and CRF3. PIL5 directly represses the expression of these *CRF* genes, suggesting a direct effect of PIL5 on CTK responsiveness in *Arabidopsis* (Oh *et al*., 2009). However, it remains unclear whether PIFs involves in seed development via CTK signal transduction.

In rice, six *phytochrome‐interacting factor‐like* genes (*OsPILs*) have been identified and designated as *OsPIL11* to *OsPIL16* (Nakamura *et al*., 2007). Although most PIF functions and regulatory networks in *Arabidopsis* have been characterized, little is known about their physiological roles in rice. Previous studies have shown that the overexpression of *OsPIL13* in transgenic rice plants promotes internode elongation (Todaka *et al*., 2012), and the *ospil13* mutant results in pale green leaves with significantly reduced levels of chlorophyll (Chl) and Chl‐binding proteins (Sakuraba *et al*., 2017). Seedling growth in rice plants overexpressing *OsPIL15* is also found to be repressed under dark conditions (Zhou *et al*., 2014). Moreover, OsLF was found to interact with OsPIL13 and OsPIL15 to repress rice flowering (Zhao *et al*., 2011). Although OsPIL16 is a known negative regulator of rice grain length and weight (Heang and Sassa, 2012c), the functions and regulatory networks of *OsPIL15* in rice, especially in rice grains, are still unclear. In this study, we therefore created knockout (KO) *OsPIL15* lines using CRISPR/Cas9. We then used the endosperm‐specific *Gt13a* promoter to overexpress (OX) and suppress *OsPIL15*. The molecular mechanisms underlying the regulatory response in rice grains are subsequently discussed in terms of improvements in growth.

## Results

### Expression and characterization of *OsPIL15* in rice

**Figure 1:**
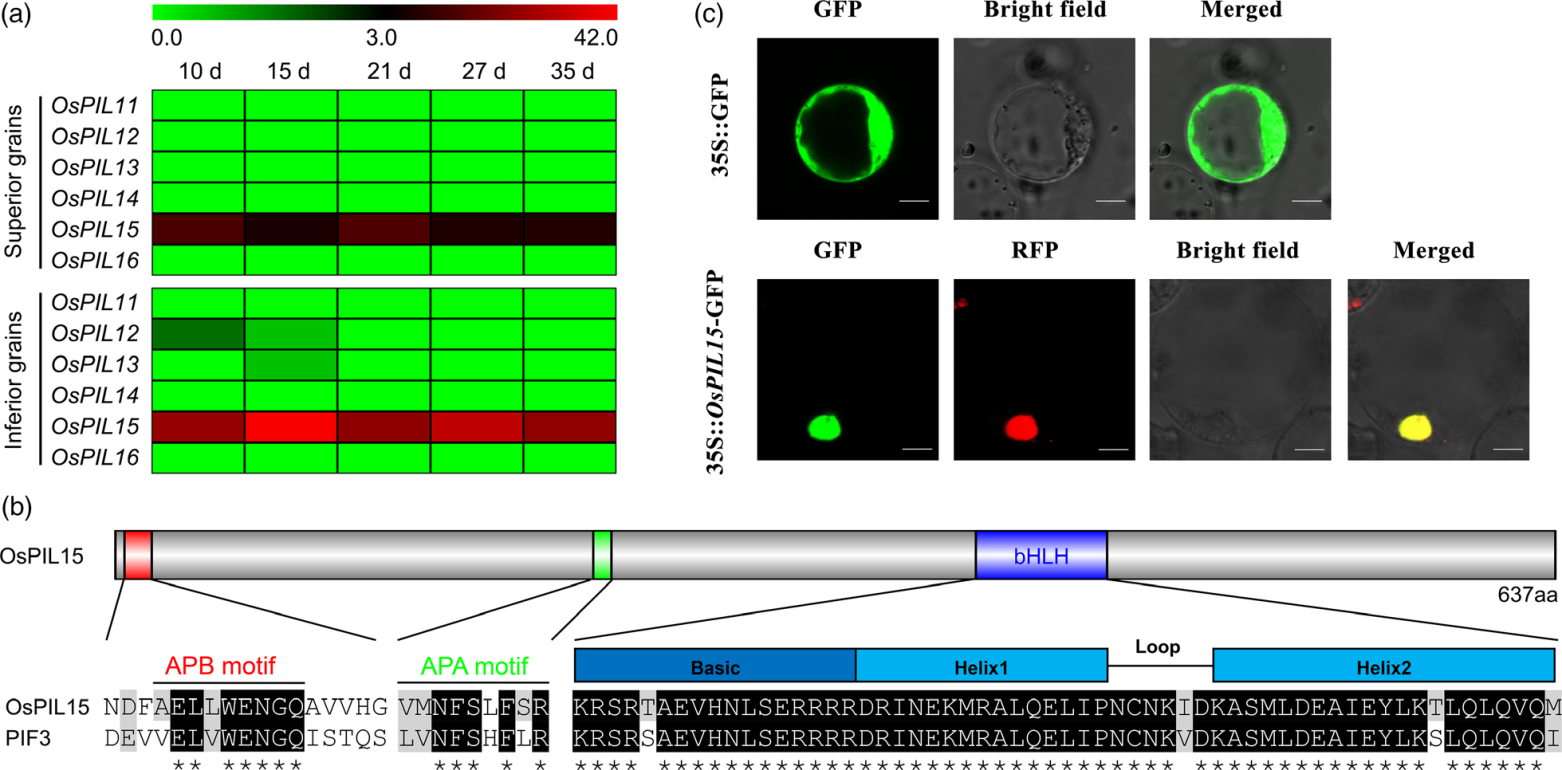
Expression patterns of *OsPILs* in rice grains and characterization of *OsPIL15*. (a) Heatmaps shows the expressional levels of *OsPILs* family members in superior and inferior grains. Expression is measured by transcripts per million clean tags (TPM). Days (d) represents days after flowering (DAF). (b) Diagram representation of the OsPIL15 protein and multiple sequence alignment of OsPIL15 domains with *Arabidopsis* PIF3. Specific sites of active phytochrome B binding (APB), active phytochrome A binding (APA) and the basic helix‐loop‐helix domain (bHLH) are presented. Conserved amino acids are shown with a gray background. Asterisks indicate fully conserved amino acids. (c) Subcellular localization of the OsPIL15 protein. The 35S::GFP and 35S::*OsPIL15*‐GFP plasmid were transformed in rice protoplast cells respectively. Red fluorescence signals were used as a nuclear marker. Scale bars: 6 μm.

To understand the role of *OsPILs* in rice grains, we first analyzed the expression pattern of them. In our laboratory preliminary study, superior and inferior rice grains were separated from the panicle at 10, 15, 21, 27 and 35 days after flowering (DAF; Sun *et al*., 2015). Thus, we analyzed the dynamic expression levels of six *OsPIL* genes in superior and inferior rice grains at the whole transcriptome level using RNA‐Sequencing (RNA‐Seq). Of these, *OsPIL11*,* OsPIL14* and *OsPIL16* expression was not detected, *OsPIL12* expression was detected in inferior grains at 10 and 15 DAF, *OsPIL13* expression was detected in inferior grains at 15 DAF, and only *OsPIL15* expression was detected in both superior and inferior grains. The expression patterns of *OsPIL15* showed that inferior grains expressed higher levels than superior grains at all five time points (Figure 1a), suggesting that *OsPIL15* may play an important role during rice grain development. So we decided to investigate the function of *OsPIL15* in regulating rice grains. Then, a phylogenetic analysis of PIFs from rice and *Arabidopsis* was performed using ClustalW (Thompson *et al*., 2003) and MEGA 6 (Tamura *et al*., 2013) software. Based on a comparison of homologous amino acid sequences, OsPIL15 was found to share a close genetic relationship with OsPIL16 from rice and PIF3 from *Arabidopsis* (Figure S1). A subsequent analysis of the OsPIL15 protein sequence revealed that outside the highly conserved bHLH domain, it also contained two characteristic domains, an active phytochrome B (APB) motif and an active phytochrome A (APA) motif (Figure 1b).

To achieve the subcellular localization of OsPIL15, an *OsPIL15*‐GFP (green fluorescent protein) fusion protein was constructed under the control of the constitutive *Cauliflower mosaic virus* (CaMV)‐35S promoter. During transient expression of the fusion protein in rice protoplasts, green fluorescence was visible throughout the cytoplasm in protoplasts with the 35S::GFP control plasmid. In protoplasts transiently expressing 35S::*OsPIL15*‐GFP, nuclear localization of the *OsPIL15*‐GFP fusion protein was revealed by colocalization with red fluorescent protein (RFP) used as a nuclear localization marker. The alignment of the predominant red fluorescence in the nucleus with the GFP fluorescence signals (Figure 1c) confirmed the localization of OsPIL15 in the nucleus.

### Regulating function of *OsPIL15* on grain size and yield

We subsequently generated single‐guide RNA (sgRNA; bases 369–388 from the ATG start codon in the cDNA) constructs then introduced them into rice to knockout the *OsPIL15* gene using CRISPR/Cas9 (Figure S2a, d). We obtained three independent homozygous KO plants (T_2_ generation). Lines KO‐1 (1‐base G insertion) and KO‐6 (1‐base T insertion) represented frameshift mutants of *OsPIL15* in the wild‐type (WT), while KO‐3 harbored a deletion of 66 bases, leading to amino acid deletions but no frame shift (Figures S2e and S3). To further analyze the function of *OsPIL15* during rice seed development, we overexpressed and suppressed *OsPIL15* using the *Gt13a* promoter (Figure S2b–c), which is stringently endosperm‐specific (He *et al*., 2011; Ning *et al*., 2008). Because the synthetic *OsPIL15* gene was not expressed in WT plants, semi‐quantitative RT‐PCR of endogenous *OsPIL15* expression in the rice endosperm at 12 DAF was used to confirm that the *OsPIL15*‐OX lines had the same level of endogenous *OsPIL15* expression as the WT. However, the synthetic *OsPIL15* gene was found to be highly expressed in all four *OsPIL15*‐OX lines (Figure S2f). As expected, the expression of *OsPIL15* in the rice endosperm was suppressed effectively in the three RNAi lines compared with the WT (Figure S2g). Independent and representative T_4_ generation plants of the OX (OX‐23, OX‐26, OX‐30 and OX‐31) and RNAi lines (RNAi‐38, RNAi‐39 and RNAi‐40) were subsequently selected for phenotype and functional analysis.

**Figure 2:**
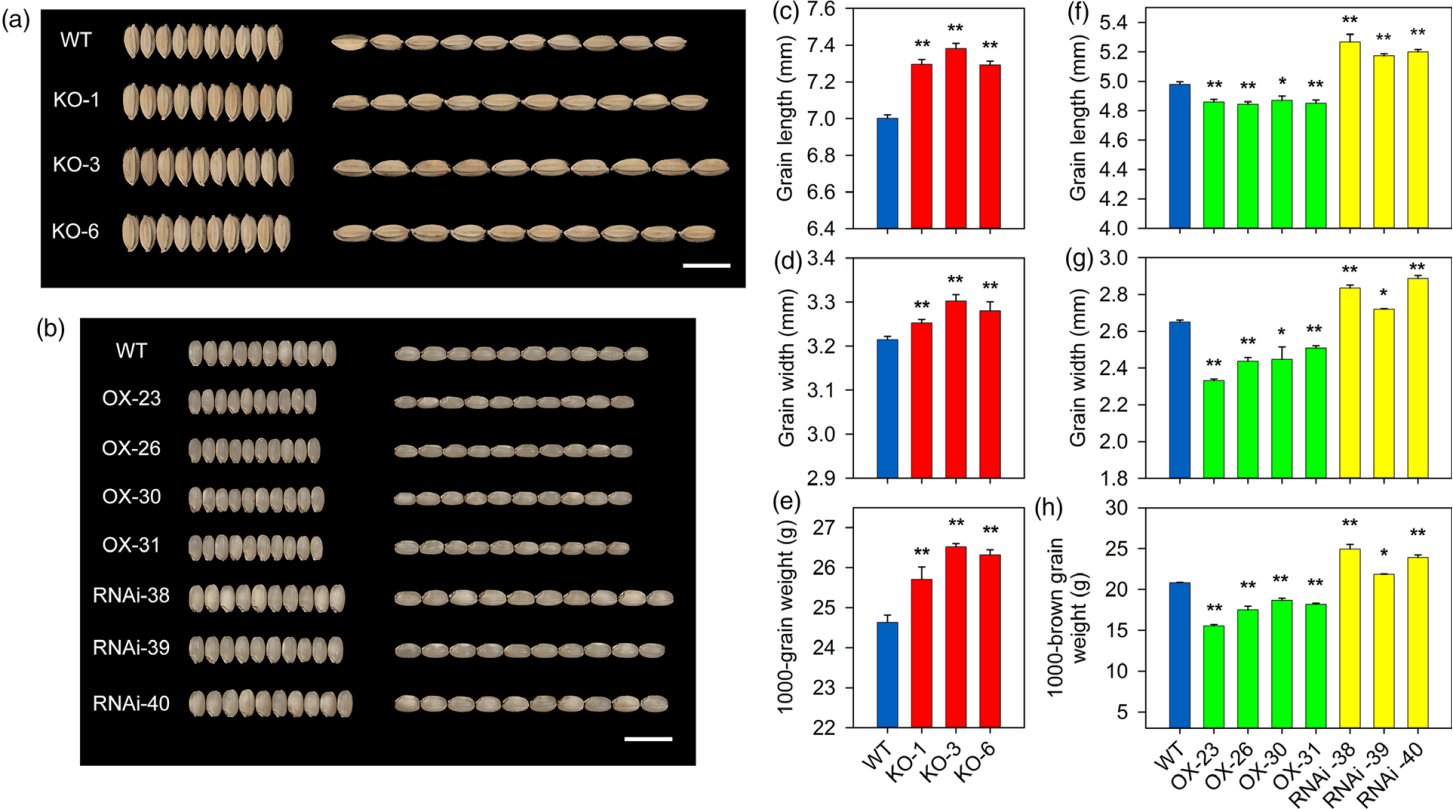
Effects of *OsPIL15* on grain size and weight. (a) Comparisons of grains between wild‐type (WT) and knockout (KO) lines. Scale bar: 1 cm. (b) Comparisons of grains between brown rice of WT, overexpressing (OX) and RNAi lines. Scale bar: 1 cm. (c–e) Grain length, grain width and 1000‐grain weight in the WT and *OsPIL15*‐KO plants. (f–h) Grain length, grain width and 1000‐brown grain weight in brown rice of WT,* OsPIL15*‐OX and *OsPIL15*‐RNAi plants. All data represent means ± SEM (*n* = 5). * *P *<* *0.05, ** *P *<* *0.01.

To investigate the effect of the altered *OsPIL15* expression on grain size, we evaluated grain length, grain width and the 1000‐grain weight. The *OsPIL15*‐KO lines were found to display a substantial increase in grain length and width compared to the WT, contributing to a significant increase in grain weight (Figure 2a, c–e). This was also consistent with previous generation (T_1_) data sets (Table S1). Morphological differences were also observed between WT and KO lines at the vegetative stage in consecutive years. The *OsPIL15*‐KO lines and WT had the same tiller number; however, reductions in plant height were observed in the KO lines (Figure S4a, Table S2). The use of the endosperm‐specific *Gt13a* promoter resulted in no significant differences in plant height and tiller number between the *OsPIL15*‐OX and *OsPIL15*‐RNAi lines and WT (Figure S4b–c, Table S2). Mature rice grain hulls were subsequently shelled to examine the endosperm phenotype. Grain sizes of brown rice, including grain length and width, were also observed. The *OsPIL15*‐OX lines showed a significant reduction in grain length, grain width and the 1000‐grain weight of brown rice, whereas the *OsPIL15*‐RNAi lines showed a significant increase in these three parameters (Figure 2b, f–h). Similar results were obtained for the T_2_ and T_3_ generations (Table S1). In addition, the grain yield of WT and transgenic lines was measured in field plots. Compared to WT, the yield in *OsPIL15*‐KO and *OsPIL15*‐RNAi lines increased by 13.07%–16.59% and 14.33%–22.08%, respectively, while it significantly decreased in *OsPIL15*‐OX plants (Table S3).

### *OsPIL15* regulates grain size by controlling cell division

**Figure 3:**
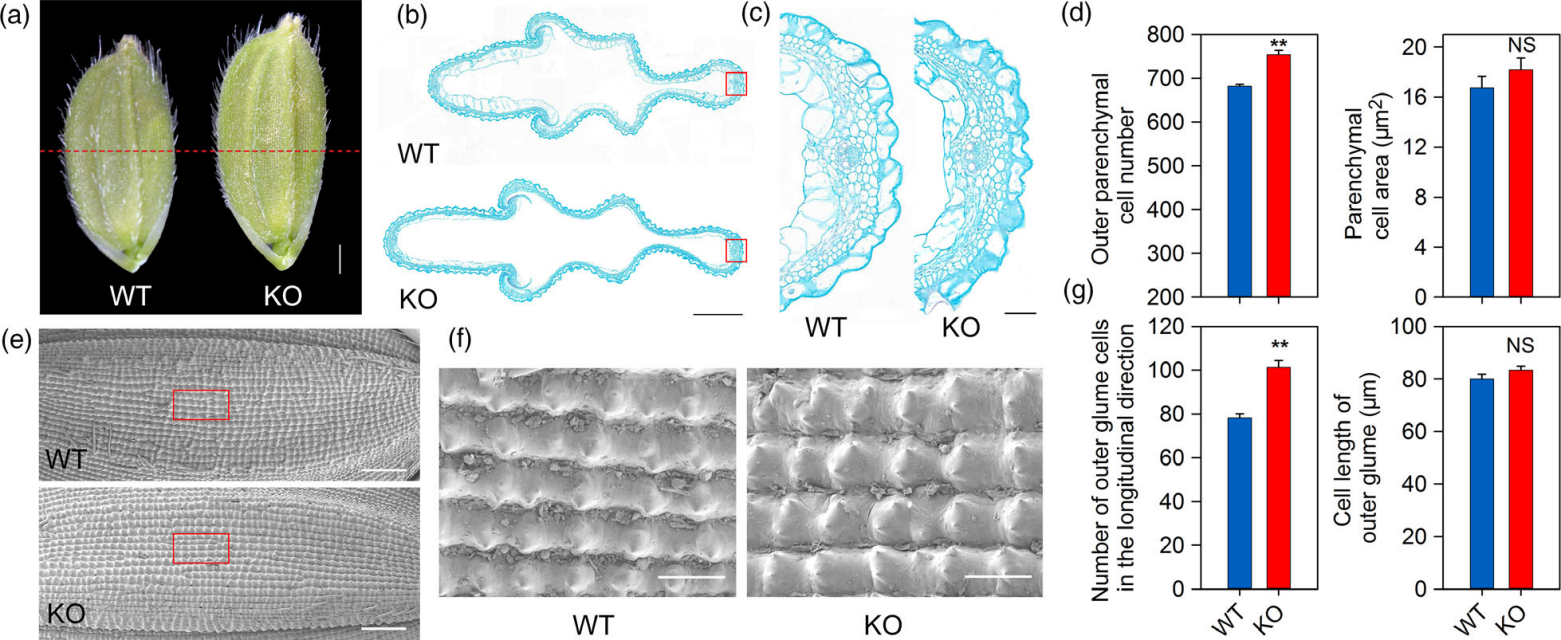
Histological analysis of spikelet hulls. (a) Young spikelet hulls of wild‐type (WT) and knockout (KO) lines. The red dashed line indicates the cross‐section position. Scale bar: 1 mm. (b) Cross‐sections of spikelet hulls of WT and KO lines. Scale bar: 500 μm. (c) Magnified view of the cross‐section area boxed in (b). Scale bar: 50 μm. (d) Cell numbers and cell areas in the outer parenchyma layer of the spikelet hulls of WT and KO lines. ** *P *<* *0.01. (e) Scanning electron photographs of the outer glume of mature seeds of WT and KO lines. Scale bars: 500 μm. (f) Magnified view of the outer surface area boxed in (e). Scale bars: 100 μm. (g) Cell numbers and cell lengths of fully mature seeds along the longitudinal axis. ** *P *<* *0.01, NS: not significant.

Mature rice grains are mainly composed of a spikelet hull and endosperm, with the spikelet hull restricting overall grain size. To confirm the role of cell number and size in the increased grain size of KO lines, histological cross‐sections of the spikelet hulls were analyzed just before flowering. The grain size of the spikelet hull was found to be larger in the KO lines than in the WT (Figure 3a). Careful examination revealed a significant increase in the number of parenchyma cells in the KO lines. The cell area of the KO lines exhibited only a slight increase, with no significant difference when compared with the WT (Figure 3b–d). These findings suggest that the change in size in the KO lines was mainly the result of a change in cell number rather than cell size. To further examine grain size in the KO lines, cell number and cell length were determined in the outer epidermis of mature seeds. More cells were observed in the lemma along the longitudinal axis in *OsPIL15*‐KO lines compared with the WT, suggesting that the increase in grain length in the KO lines was mainly the result of an increase in cell number, not cell expansion (Figure 3e–g). Cross‐sections of endosperm from WT, *OsPIL15*‐OX and *OsPIL15*‐RNAi lines were subsequently compared to determine the differences in endosperm cells. The cross‐sectional area of endosperm was significantly larger in RNAi lines but smaller in OX lines than those of WT (Figure S5a, c). However, there was no significant difference in the number of endosperm cells per unit area (Figure S5d), and the average endosperm cell area was calculated with no significant difference. These results suggest that more endosperm cells are required to fill the endosperm of *OsPIL15*‐RNAi lines compared to *OsPIL15*‐OX lines (Figure S5e). Consistently, the area of the outermost layer of the endosperm, the aleurone, was also compared, and no significant difference was observed in average aleurone cell area (Figure S5f). Taken together, these findings suggest that *OsPIL15* is involved in the regulation of grain size by controlling cell division.

### Identification of genes regulated by *OsPIL15*

**Figure 4:**
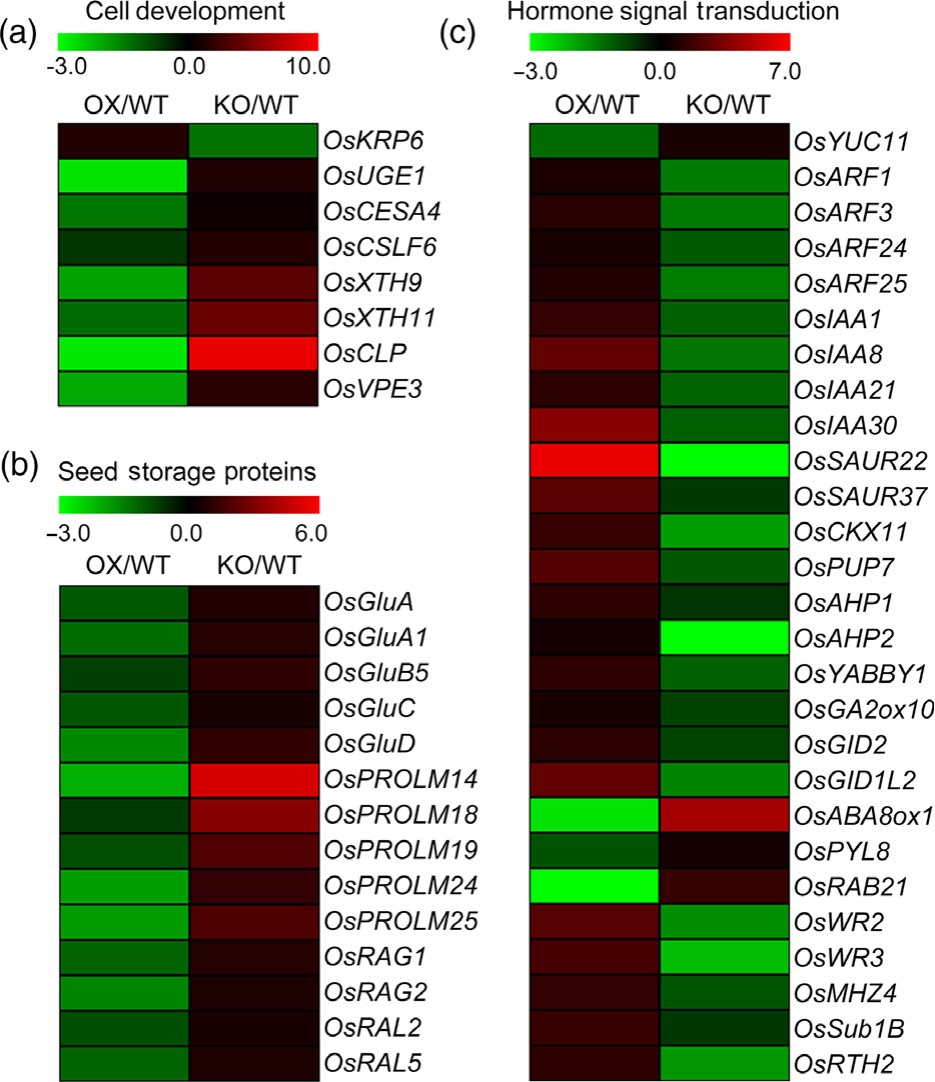
Expression levels of various genes regulated by *OsPIL15*. Heatmaps showing the fold changes in expression of gene involved in (a) cell development, (b) seed storage proteins and (c) hormone signal transduction. Log_2_ (fold changes) are represented by a colour scale from green (down‐regulated expression) to red (up‐regulated expression).

To further explore the mechanism of *OsPIL15* on grain size at the genetic level, RNA‐Seq analysis was carried out using endosperm samples obtained 12 DAF from WT, *OsPIL15*‐KO and *OsPIL15*‐OX lines to compare the global transcriptional profiles of differentially expressed genes (DEGs). For each sample were calculated based on the normalized expression results, revealing correlation coefficients of 0.88/0.94 and 0.92/0.84 for the WT/*OsPIL15*‐OX and WT/*OsPIL15*‐KO plants respectively. We identified 6262 and 8666 DEGs in the OX versus WT (OX/WT) and KO versus WT (KO/WT) respectively. To validate the expression profiles, qRT‐PCR analysis was performed using several randomly selected genes (Figure S6). The results suggested that RNA‐Seq was an accurate and reliable method of identifying DEGs in the WT, *OsPIL15*‐KO and *OsPIL15*‐OX lines. Such large changes in the transcript levels of the genes involved in various pathways suggest that *OsPIL15* triggers a change in complex regulatory networks in the rice grains. Gene ontology (GO) analysis was therefore carried out to further understand each DEG (Figure S7). Of all the DEGs, a total of 3,446 genes were detected in both OX/WT and KO/WT. We further studied the genes with opposite expression pattern between OX/WT and KO/WT. In our RNA‐Seq data, eight DEGs involved in cell development were identified (Figure 4a, Table S4). Fourteen genes controlling rice seed storage proteins were detected. Of these, five corresponded to glutelins, five to prolamins and four to allergenic proteins, and all were up‐regulated in the *OsPIL15*‐KO lines (Figure 4b, Table S4). Then, we detected 27 genes involved in hormone signal transduction including auxin, CTK, gibberellic acid (GA), abscisic acid (ABA) and ethylene (ETH; Figure 4c, Table S4).

### OsPIL15 directly targets *OsPUP7*

Transcription factors regulate gene expression by binding to their promoters, thereby promoting or inhibiting their subsequent transcription. A previous study revealed that PIF3 binds to the G‐box (CACGTG) or PBE‐box (CACATG), while OsPIF14 and OsPIL16 bind to the N‐box [CACG(C/A)G] (Cordeiro *et al*., 2016; He *et al*., 2016; Zhang *et al*., 2013). Based on these findings, an electrophoresis mobility shift assay (EMSA) was performed to identify the specific DNA sequences to which OsPIL15 binds. Purified OsPIL15‐His protein was obtained from *Escherichia coli* and then four probes corresponding individually to the G‐box, PBE‐box, N1‐box (CACGCG) and N2‐box (CACGAG) were designed (Table S5). The results showed strong signals between OsPIL15 and the N1‐box, but weak signals with the PBE‐box. No signals were observed with the G‐box or N2‐box probes (Figure S8a). Next, using the DEGs listed in Table S4, a 3‐kb region upstream of the transcription start site was selected as the gene promoter region to determine the location of the N1‐box. Of the 49 DEGs, 13 genes contained the N1‐box (Table S4) and of these, eight (*OsUGE1*,* OsXTH9*,* OsXTH11*,* OsARF3*,* OsARF25*,* OsPUP7*,* OsSAUR22* and *OsRAB21*) were selected to examine the binding ability of the N1‐box to OsPIL15 (Figure S8b). Compared with the negative control and free probes, the combination of OsPIL15‐His and the *OsPUP7* probe displayed a slower migrating interaction, with no binding bands detected in the other probe reactions (Figure S8c).

**Figure 5:**
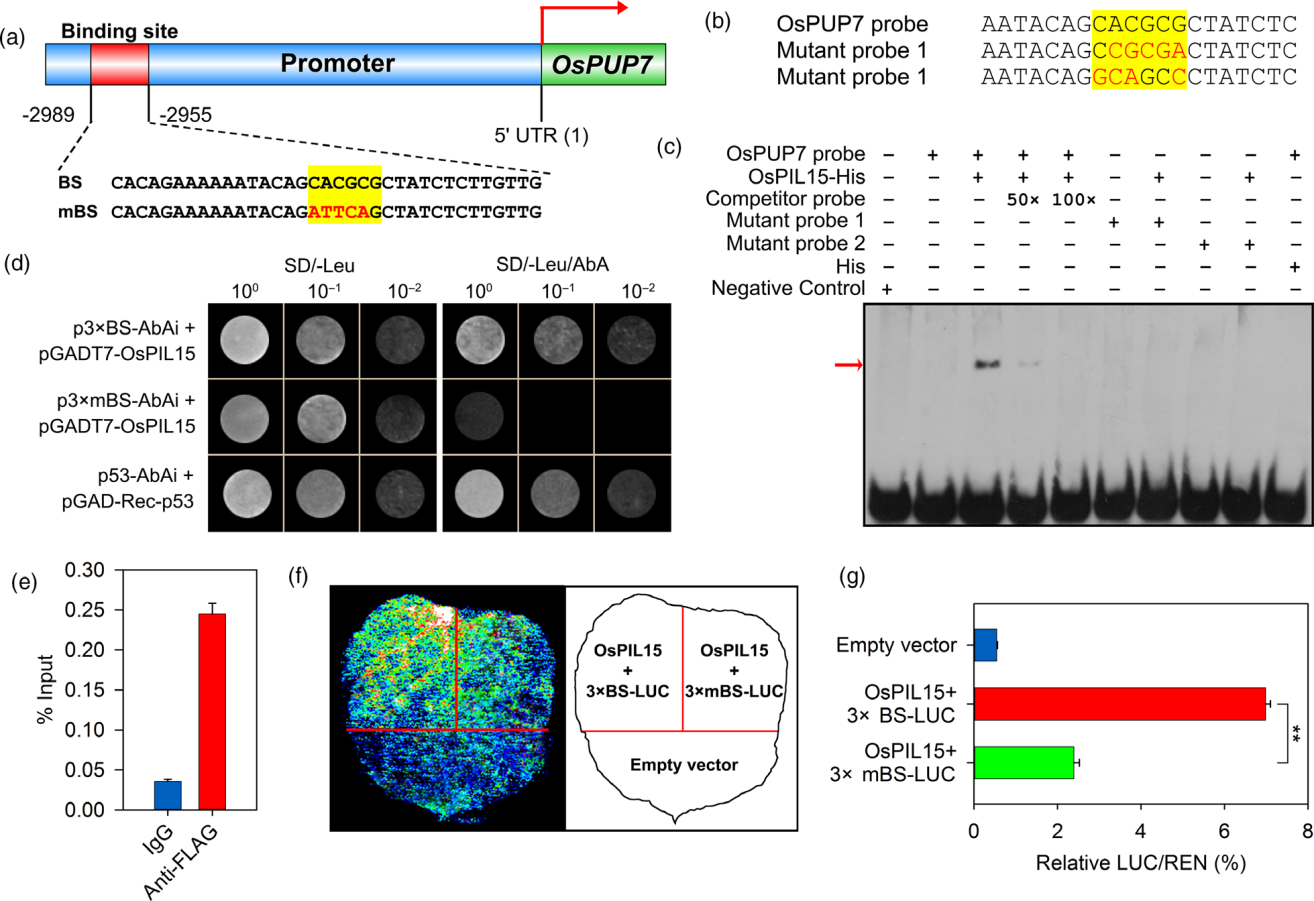
Direct targeting of *OsPUP7* by OsPIL15. (a) Schematic representation showing localization of the putative OsPIL15 binding site (BS) in the *OsPUP7* promoter. Yellow highlighting denotes the N1‐box. Mutated nucleotides are shown in red in the mutated binding site (mBS). (b) Sequences of the *OsPUP7* and corresponding mutant probes used in the electrophoresis mobility shift assay (EMSA). Yellow highlighting denotes the N1‐box. Mutated nucleotides are shown in red. (c) EMSA assay of binding between OsPIL15 and the target gene *OsPUP7*. The competitor probe was added at 50‐ and 100‐fold more than the labeled probes respectively. A negative control was used to validate the EMSA system. The red arrow indicates the position of the shifted bands. (d) Yeast one‐hybrid assays of OsPIL15 binding to the *OsPUP7* promoter. Constructs pGAD‐Rec‐p53 and p53‐AbAi were used as positive control. (e) ChIP‐qPCR analysis of OsPIL15 binding to the promoter of *OsPUP7 in vivo*. DNA samples obtained before immunoprecipitation were used as the input, with IgG as a negative control. Data were normalized with input transcripts. (f) Transcriptional activation of *OsPUP7* expression by OsPIL15 using the dual‐luciferase (LUC) system in *N. benthamiana* leaves. (g) Quantification of LUC activity shown in (f). Relative renilla (REN) luciferase activity was used as an internal control. Data represent means ± SEM (*n* = 6). ** *P *<* *0.01.

Analysis of the *cis*‐elements of the putative *OsPUP7* promoter [3 kb upstream of the 5′ untranslated region (UTR)] revealed a putative OsPIL15 binding site at −2989 to −2955 bp upstream of the transcription start site (Figure 5a). We first evaluated the binding specificity of OsPIL15 to the N1‐box sequence of the *OsPUP7* promoter using binding competition assays. An unlabeled competitor probe and two labeled mutant probes were used for the EMSA (Figure 5b). As shown in Figure 5c, OsPIL15 specifically bound to *OsPUP7* promoter fragments containing a normal N1‐box sequence, but did not bind to the promoter fragments with mutated N1‐box sequences. In the competition reaction, unlabeled cold probes were mixed at 50‐ and 100‐fold excess with the labeled probes, which revealed a drastic signal reduction in the probe shift band with 50‐fold excess of unlabeled cold probe; however, no singles were observed with the addition of 100‐fold excess unlabeled cold probes. Fifty‐fold unlabeled cold probe‐binding activity was therefore quantified by densitometry analysis of EMSA films using ImageJ software, there was an approximately 88.63% reduction in probe‐binding activity with biotin compared to the probe‐binding activity of the *OsPUP7* probe. These results suggest that the binding was specific.

Then to examine the direct regulation of OsPIL15 by the *OsPUP7* promoter, yeast one‐hybrid (Y1H) assay was carried out. The pGAD‐Rec‐p53 and p53‐AbAi constructs were used as a positive control. The pGADT7‐*OsPIL15* and p3×BS‐AbAi transformants, but not the pGADT7‐*OsPIL15* and p3×mBS‐AbAi transformants, grew well on the SD/‐Leu medium with AbA, suggesting that OsPIL15 specifically binds to the binding site of the *OsPUP7* promoter (Figure 5d). To further determine whether OsPIL15 directly associates with the promoter of *OsPUP7 in vivo*, a chromatin immunoprecipitation (ChIP) assay was performed. We observed that the promoter fragments of *OsPUP7* were greatly enriched in the chromatin fractions by anti‐FLAG antibody compared to that by control IgG (Figure 5e). In addition, luciferase was used as a reporter to evaluate the effect of OsPIL15 on *OsPUP7* expression. Cotransformation of the binding site in *OsPUP7* promoter and OsPIL15 showed that OsPIL15 significantly enhances transcriptional activation of *OsPUP7* compared with co‐expression of OsPIL15 and mutated binding elements of *OsPUP7* (Figure 5f–g). Taken together, these findings confirmed that OsPIL15 positively regulates expression of *OsPUP7* by directly binding to its promoter region.

### OsPIL15 controls cell division via *OsPUP7*

qRT‐PCR analysis further indicated that the expression levels of *OsPUP7* in the *OsPIL15*‐KO and *OsPIL15*‐RNAi lines were significantly lower than those in the WT. In contrast, *OsPUP7* expression significantly increased in *OsPIL15*‐OX lines (Figure S9b–d). *OsPUP7* is thought to be involved in the selective transport of isopentenyl adenine (iP) and isopentenyl adenosine (iPA; Qi and Xiong, 2013). To determine levels of iP and iPA in spikelets of the *OsPIL15*‐KO lines, contents were measured using high‐performance liquid chromatography‐tandem mass spectrometry (HPLC‐MS/MS). No iP was detected in spikelets of WT or *OsPIL15*‐KO lines, suggesting that extremely low iP levels were present; however, the iPA content was higher in the *OsPIL15*‐KO lines than that in WT (Figure S9a).

**Figure 6:**
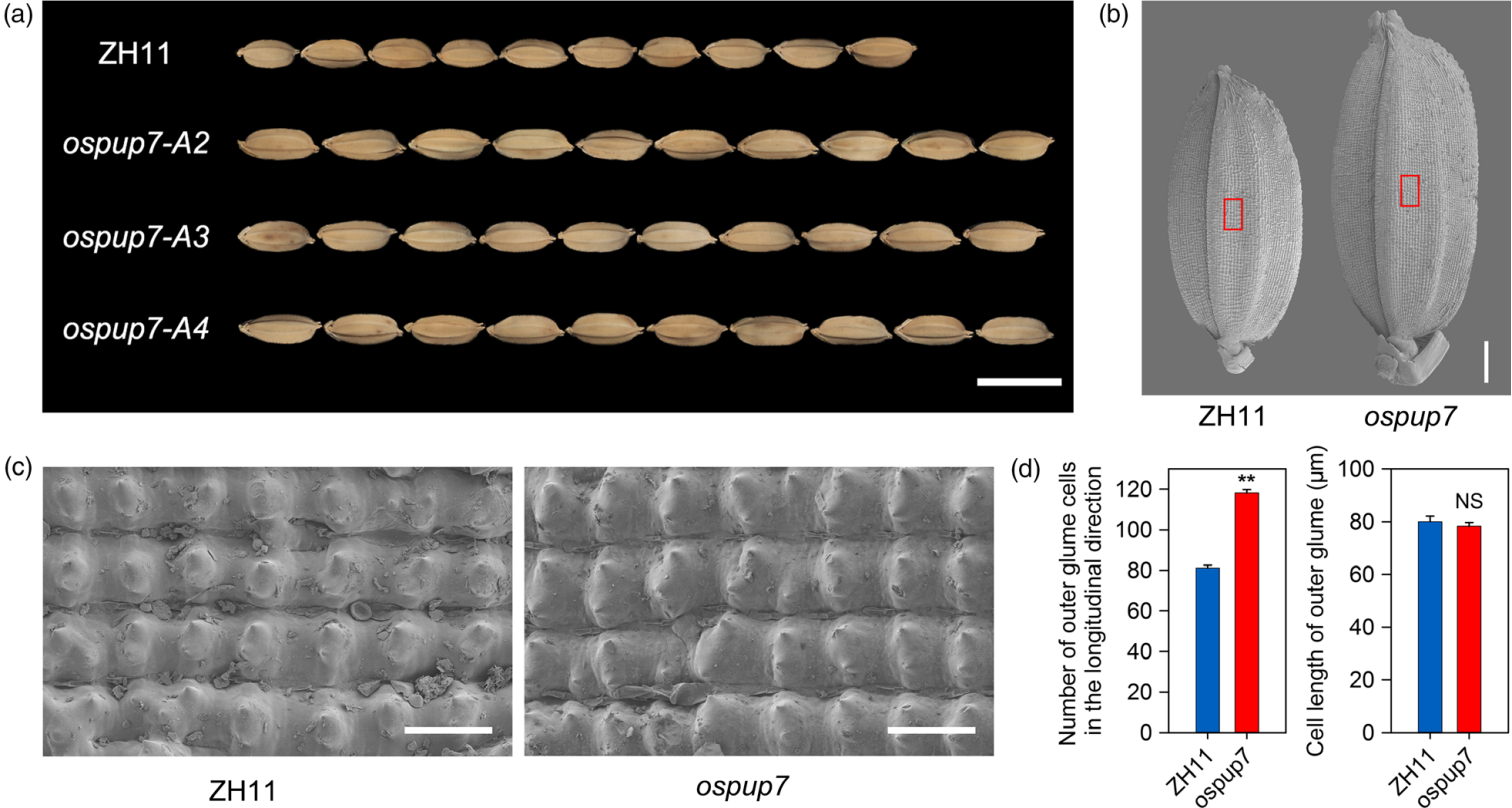
Comparison of mature grains between the *ospup7* mutant and wild‐type Zhonghua11 (ZH11). (a) Grains of ZH11 and the *ospup7* mutant. Scale bar: 1 cm. (b) Scanning electron photographs of the outer glume of mature seeds of ZH11 and the *ospup7* mutant. Scale bars: 1 mm. (c) Magnified view of the outer surface area boxed in (c). Scale bars: 100 μm. (d) Cell numbers and cell lengths of fully mature seeds along the longitudinal axis. ** *P *<* *0.01, NS: not significant.

With the end to clarify whether *OsPUP7* also regulates cell division, we obtained *ospup7* mutants (Qi and Xiong, 2013). As previously reported, the grain length of the mutant was significantly longer than that of WT Zhonghua 11 (ZH11; Figure 6a–b). Meanwhile, scanning electron microscopy showed a significant increase in cell number in the lemma along the longitudinal axis in *ospup7* mutants compared with ZH11. However, no difference in longitudinal cell length on the outer surface of the glume was observed between the *ospup7* mutants and ZH11 (Figure 6c–d). These results suggest that the longer grain length phenotype of the *ospup7* mutants is the result of an increase in longitudinal cell numbers in the spikelet hulls. Overall, these findings further confirm that the OsPIL15‐*OsPUP7* module participates in the regulation of grain size, at least in part, by modulating cell division.

## Discussion

Phytochrome‐interacting factors regulate a number of pathways including seed germination, the circadian clock, flowering time, stomatal development and senescence, suggesting that they act as signaling hubs during the regulation of plant growth and development (Jeong and Choi, 2013; Leivar and Monte, 2014). Although the physiological functions and underlying molecular mechanisms of PIFs have been studied extensively in *Arabidopsis*, little is known about PIFs in rice, especially in regulating rice grain size. In the present study, we constructed transgenic rice (KO, OX and RNAi lines) to examine the role of *OsPIL15* in regulating grain size. Inhibiting expression of OsPIL15 increased grain size and improved rice yield (Figure 2, Table S3). The *OsPUP7* gene, which encodes purine permease, was subsequently screened from 49 DEGs as the target gene of OsPIL15 (Figure S8). We demonstrated that the CACGCG element (N1‐box) in the *OsPUP7* promoter was directly bound by OsPIL15 using EMSA assay and Y1H experiment *in vitro*, also ChIP‐qPCR detection *in vivo* (Figure 5). Furthermore, mutation of *OsPIL15* led to a decrease in *OsPUP7* expression, while overexpression of *OsPIL15* caused an increase (Figure S9). Dual‐luciferase assays also revealed the enhancing effect of OsPIL15 on transcriptional activity of *OsPUP7* (Figure 5f–g). Meanwhile, *ospup7* mutants with larger grain lengths have been reported (Qi and Xiong, 2013). Therefore, these findings suggest that the OsPIL15‐*OsPUP7* module plays important roles in regulating rice grain size.

Cytokinin plays a crucial role in regulating the proliferation and differentiation of plant cells, as well as various growth and development processes (Schaller *et al*., 2014; Werner and Schmulling, 2009). It has been reported that simultaneous disruption of the three CTK receptors (*ahk2 ahk3 ahk4* triple mutant) results in a smaller shoot and root apical meristem due to reduced cell division (Riefler *et al*., 2006). Moreover, young spikelets are a major source of CTK synthesis. Cell number and cell division activity, which subsequently affect seed size, are also regulated by levels of CTK (Jameson and Song, 2016; Yang *et al*., 2002). *OsMADS29* is thought to play a role in auxin/CTK homeostasis, affecting the size and number of starch granules as well as the size of endosperm cells (Nayar *et al*., 2013). In this study, we confirmed that OsPIL15 regulates grain size by affecting cell number (Figure 3, Figure S5). Mutations in one of its target genes, *OsPUP7*, further validated its function in regulating cell proliferation (Figure 6). A previous study revealed that *OsPUP7* has a negative effect on CTK transport and that transport was selective, with *ospup7* mutant spikelets accumulating higher levels of iP and iPA than WT plants (Qi and Xiong, 2013). We also found iPA accumulation in the spikelets of *OsPIL15*‐KO compared with WT plants (Figure S9a). Therefore, these findings suggested that OsPIL15 functions on determining grain size by regulating cell division, at least partially directly targeting *OsPUP7* and thereby inducing CTK transport.

The phylogenetic tree of OsPIL15 homologs showed that OsPIL15 shares a close genetic relationship with OsPIL16 (Figure S1). OsPIL16 negatively regulates grain length by controlling cell elongation in the lemma/palea, while its function is inhibited by OsPGL1 and OsPGL2 via heterodimerization (Heang and Sassa, 2012a,2012b,2012c). However, OsPIL15 was found to be a negative regulator of grain size via its effect on cell division. The Y2H data further suggest that OsPIL15 interacts weakly with OsPGL1, with no interaction with OsPGL2 (Figure S10). Therefore, OsPIL15 and OsPIL16 play roles in regulating grain size, but are thought to act independently, with OsPIL15 participating in its own complicated molecular regulatory network. As members of the bHLH transcription factor superfamily, PIFs are able to regulate the expression of a large number of genes directly or indirectly, thereby mediating various physiological and developmental processes (Jeong and Choi, 2013). Grain size is controlled by a complex genetic network, with some of the genes involved yet to be identified (Gao *et al*., 2013). Using the *OsPIL15*‐KO and *OsPIL15*‐OX lines, we identified a number of genes involved in the regulation of cell development, seed storage proteins and hormone signal transduction (Figure 4, Table S4). In addition, most DEGs were clustered in GO analysis of the distribution of gene function (Figure S7). However, details of this potential mechanism require further elucidation. Investigation of the functional roles of these genes in rice grain development is now needed to better understand the effects of OsPIL15 and the molecular mechanism underlying regulation of grain size in rice.

**Figure 7:**
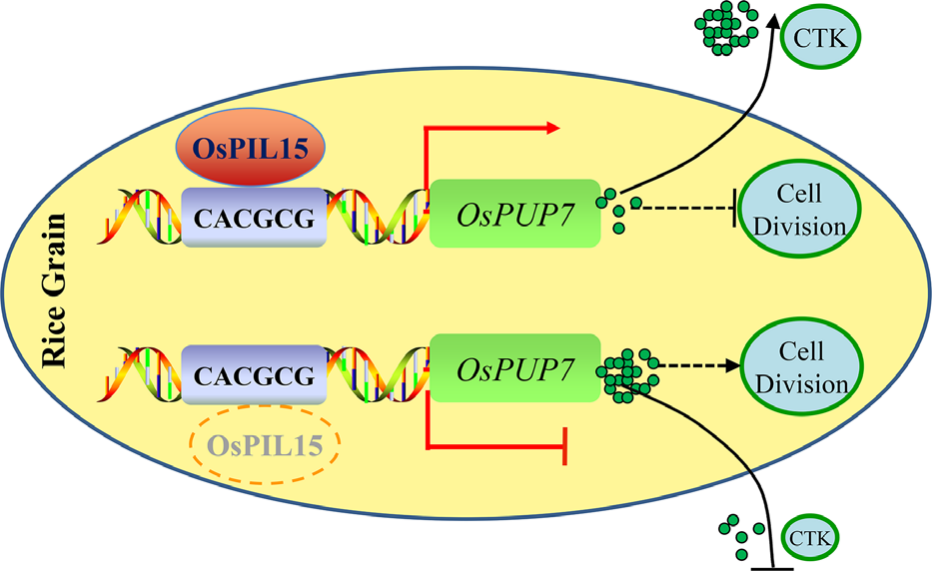
Proposed model explaining the function of OsPIL15 in regulating grain size in rice. OsPIL15 is thought to up‐regulate expression of *OsPUP7* by directly binding to the N1‐box motif (CACGCG) of the *OsPUP7* promoter to influence CTK transport. Without the OsPIL15 protein (lower diagram, denoted by a red dotted oval), expression of *OsPUP7* is inhibited, reducing CTK transport from spikelets to other tissues, thereby promoting cell division, resulting in increased grain size. Arrows indicate induction, and T‐lines indicate repressive action. Dashed lies indicate putative signaling pathways.

Based on the results of this study, we proposed a model to describe the functions of rice OsPIL15 in regulating grain size (Figure 7). OsPIL15 is thought to up‐regulate the expression of *OsPUP7* genes by binding directly to the N1‐box motif of the *OsPUP7* promoter to influence CTK transport. The *OsPIL15*‐KO lines lacking the OsPIL15 protein showed decreased expression of *OsPUP7*, reducing CTK transport from the spikelets to other tissues, thereby promoting cell division and increasing grain size. Further investigations are necessary to identify the interacting proteins of OsPIL15 in rice. This would provide a more thorough insight into the molecular functional network based on an OsPIL15‐*OsPUP7* regulatory module for rice grain size. In conclusion, the reduced expression of *OsPIL15* can increase grain size and weight (Figure 2). Therefore, inhibiting the expression of *OsPIL15* in rice should be important for improving grain yield (Table S3). CRISPR technology is an effective method for inhibiting gene expression in plants, thus, *OsPIL15* could serve as a viable candidate gene for targeted genome editing to improve grain yield. The manipulation of *OsPIL15* could help breeders develop new elite rice varieties with high yields in the future.

## Materials and methods

### Plant materials and plasmid construction

Rice (*Oryza sativa* L. cv. Nipponbare) was grown under natural field conditions at the Henan Agricultural University research farm, Henan Province, China, during the rice‐growing seasons of 2014–2018. The planting density was 13 × 30 cm, with one plant per hill. *OsPIL15*‐KO plants were constructed using CRISPR/Cas9 with the expression vector pBUN411, which was kindly provided by Prof. Qijun Chen (Xing *et al*., 2014). The sequence of the target site was 5′‐GACTTCTTCTCCGAGCTCCAGG‐3′, which contained a protospacer adjacent motif (PAM) AGG at the 3′ end and a *Sac*I site neighboring PAM. The synthesized oligos (Table S6) were annealed and inserted into the *Bsa*I sites of the pBUN411 vector.

The coding sequence (CDS) of *OsPIL15* (Os01g0286100) was obtained from The Rice Annotation Project Database (http://rapdb.dna.affrc.go.jp/), and optimized and synthesized by GENEWIZ (Suzhou, China; Table S7). The maize *ubiquitin* (*Ubi*) promoter in the pTCK303 expression vector was replaced with a strong endosperm‐specific *Gt13a* promoter. To construct the *OsPIL15*‐OX vector, the CDS was inserted into the *Kpn*I‐*Sac*I region of the pTCK303‐*Gt13a* vector. To generate the hairpin RNAi construct, a 317‐bp partial fragment of *OsPIL15* downstream of the translation start codon was amplified. *OsPIL15* antisense fragments were then released by digesting with *Kpn*I and *Bam*HI subcloned in the pTCK303‐*Gt13a* vector to generate reverse insertion. *OsPIL15* sense fragments released by digestion with *Spe*I and *Sac*I were also subcloned into the same vector to generate forward insertion.

### Subcellular localization analysis

To construct the fusion proteins, the CDS of *OsPIL15* without the termination codon was cloned into the N terminus of GFP under control of the CaMV‐35S promoter in the vector pBWA(V)HS (provided by BioRun Biotechnology Co., Ltd, Wuhan, China). The recombinant vector was then transformed into rice protoplasts as described previously (Miao and Jiang, 2007). GFP signals were visualized using a confocal laser‐scanning microscope (Olympus FluoView FV1000, Olympus, Tokyo, Japan). NLS‐mKate, a nucleus marker far‐red fluorescent protein (mKate) with a N‐terminal nucleus‐localization sequence (NLS), was used.

### Analysis of rice grain phenotypes

Harvested grains were air‐dried and stored at room temperature. Thirty fully filled grains from transgenic and WT plants were then chosen at random, and their length and width were determined using a rice appearance quality detector (JMWT12, Dong Fu Jiu Heng, Beijing, China) with five replications each. The 1000‐seed weights were calculated from the weight of 500 fully fertile seeds.

### Histological analysis

For histological analysis, young spikelet hulls, freshly picked 4 days before heading, were fixed in formalin‐acetic acid‐alcohol (FAA) solution [50% alcohol: formalin (37%–40% formaldehyde): glacial acetic acid; 18:1:1] for at least 16 h. For observations of endosperm cells, developing endosperms were sampled at 7 DAF then fixed overnight in 2.5% glutaraldehyde (w/v). After dehydration in an ethanol series, the samples were embedded in Paraplast Plus (Sigma) and LR White resin (London Resin, Berkshire, UK) respectively. Tissue sections (10 μm) were stained with safranin T and fast green and semi‐thin sections (1.5 μm) were stained with toluidine blue then observed under a light microscope (80I, Nikon, Kanagawa, Japan). The outer surface of the lemma from mature seeds was observed with a scanning electron microscope (SU8010, Hitachi, Tokyo, Japan) at an acceleration voltage of 3.0 kV. The number of cells per sample was determined, and the area of cells in each sample was obtained using Image‐Pro Plus 6.

### RNA extraction and qRT‐PCR assays

Total RNA was extracted using TRIzol reagent (Invitrogen, Carlsbad, CA) according to the manufacturer's instructions and then reverse transcribed using a reverse transcriptase enzyme (Promega, Madison, WI). RT‐PCR was performed on a CFX 96 Real‐Time System RT‐PCR system (Bio‐Rad, Hercules, CA) using GoTaq^®^qPCR Master Mix (Promega, Madison, WI) according to the manufacturer's instructions. Relative expression levels were determined using the 2^−ΔΔCT^ method (Livak and Schmittgen, 2001). The primers used are listed in Table S6.

### RNA‐Seq analysis

Samples were collected from the endosperm of WT, *OsPIL15*‐OX and *OsPIL15*‐KO plants at 12 DAF. cDNA libraries were constructed and sequenced on the BGISEQ‐500 platform at the Beijing Genomics Institute (Wuhan, China). Two biological replicates were prepared for each sequencing library. The NOISeq method was used to screen DEGs between groups (Tarazona *et al*., 2011). DEGs were selected according to the following default criteria: a fold change ≥1.5 and divergence probability ≥0.6. Data of the RNA‐Seq are available in the National Center for Biotechnology Information (NCBI) Sequence Read Archive (SRA) database, under accession BioProject ID: PRJNA503271.

### Electrophoresis mobility shift assays

Electrophoresis mobility shift assay was performed as described previously (Ma *et al*., 2009). Briefly, the CDS of *OsPIL15* was cloned into the expression vector pET28a and the constructs were transformed into *E. coli* BL21 (DE3). For purification of the fusion protein, the cells were lysed using an ultrasonic cell disruptor, and the His‐tagged fusion proteins were purified with Ni‐NTA resin (Qiagen, Valencia, CA) according to the manufacturer's instructions.

Oligonucleotides (Table S5) were synthesized and labeled using the Biotin 3′ End DNA Labeling Kit (Beyotime, Shanghai, China). DNA probes were obtained by annealing two complementary oligonucleotides. The DNA gel mobility shift assay was performed using the EMSA kit (Beyotime, Shanghai, China) following the manufacturer's protocol. Briefly, the probes were incubated for 30 min with OsPIL15 protein in binding buffer at room temperature then the DNA–protein complexes were separated on native polyacrylamide gels (6%). The results were quantified using ImageJ software.

### Yeast one‐hybrid assays

Y1H assays were used to determine binding of OsPIL15 to the *OsPUP7* promoter. The WT putative binding site (BS, CACAGAAAAAATACAGCACGCGCTATCTCTTGTTG, N1‐box underlined) of the *OsPUP7* promoter was synthesized to obtain three tandem repeat copies containing the N1‐box, and a mutated binding site (mBS, CACAGAAAAAATACAGATTCAGCTATCTCTTGTTG, mutated sequences underlined) carrying the same flanking regions but with five‐base substitutions in the N1‐box was synthesized to obtain three tandem repeat copies. Triplicate BS and triplicate mBS were subsequently synthesized and inserted into the reporter vector pAbAi respectively. Meanwhile, the CDS of *OsPIL15* was fused to the pGADT7 vector then the recombinant pGADT7‐*OsPIL15* was transformed into the Y1HGold yeast strain with the reporter linearized plasmid p3×BS‐AbAi or p3×mBS‐AbAi to determine protein–DNA interactions. Positively transformed yeast cells were determined by spotting serial dilutions (1:1, 1:10 and 1:100) of yeast onto SD/‐Leu medium supplemented with 400 ng/mL Aureobasidin A (AbA) according to the Matchmaker Gold Y1H system user manual (Clontech Laboratories Inc., Mountain View, CA). The primers used in the Y1H assay are listed in Table S6.

### ChIP‐qPCR assays

Young panicles of flowering *Ubi::OsPIL15‐3×FLAG* rice plants were cross‐linked in 1% formaldehyde then a ChIP assay was performed with technical support from GeneCreate (Wuhan, China). Immunoprecipitations were performed using an anti‐FLAG polyclonal antibody (Abclonal Biotech Co., Ltd, Wuhan, China), with normal IgG used as a negative control. The enriched DNA fragments were analyzed by qPCR. The primers used in the ChIP assays are listed in Table S6.

### Transient expression assays

The CDS of *OsPIL15* was cloned into the effector vector pGreenII 62‐SK under control of the CaMV 35S promoter (Hellens *et al*., 2005). Triplicate BS and triplicate mBS were separately inserted into the reporter vector pGreenII 0800‐LUC. Effectors and corresponding reporters were then transformed into *A. tumefaciens* GV3101 cells and co‐expressed in young *N. benthamiana* leaves. Firefly and Renilla luciferase signals were assayed using a Double‐Luciferase Reporter Assay kit (TransGen Biotech, Beijing, China). The primers used in the transient expression assay are listed in Table S6.

### Measurement of cytokinin content

The spikelets of WT and *OsPIL15*‐KO lines were used to measure CTK content (measured by Zoonbio Biotechnology Co., Ltd, Nanjing, China). To do so, samples of approximately 0.5 g were ground in a precooled mortar containing 10 mL extraction buffer (isopropanol: water: hydrochloric acid, 2:1:0.002, v/v/v). The extract was shaken at 4 °C for 30 min then 20 mL of dichloromethane was added before shaking at 4 °C for a further 30 min. The samples were then centrifuged at 17 949 ***g*** for 5 min at 4 °C then the organic phase was extracted and dried using nitrogen. The pellets were dissolved in 400 μL methanol (0.1% methane acid) and filtered using a 0.22‐μm filter membrane then the purified product was subjected to HPLC‐MS/MS analysis. HPLC analysis was performed using an ACQUITY UPLC BEH C18 (Waters, Milford, MA) column (2.1 × 100 mm; 1.7 μm) with an injection volume of 5 μL. MS was performed using a triple quadrupole Xevo TQ mass spectrometer (Waters) with the following: capillary voltage: 3.0 kV, source temperature: 150 °C, desolvation temperature: 400 °C, cone gas flow: 50 L/h, desolvation gas flow: 800 L/h. Three technical repeats per sample were performed.

### Yeast two‐hybrid assays

Split‐ubiquitin Y2H assays used to examine interactions between the proteins of OsPIL15 and OsPGL1/OsPGL2 were carried out using the DUALhunter starter kit (Dualsystems Biotech, Switzerland) according to the manufacturer's protocol. Briefly, the CDS of *OsPIL15* and *OsPGL1* (Os03g0171300)/*OsPGL2* (Os02g0747900) were cloned in frame into the pDHB1 and pPR3‐N vectors respectively. Yeast strain NMY51 cells were then cotransformed with the constructs and plated on SD/‐Leu/‐Trp (SD‐LT). Protein–protein interactions were assessed by growth of the yeast colonies on SD/‐Leu/‐Trp/‐His (SD‐LTH) and SD/‐Leu/‐Trp/‐His/‐Ade/(SD‐LTHA). Four microlitres of serial dilutions (1, 1:10, 1:100 and 1:1000) were spotted on solid medium supplemented with 5 mm 3‐AT plates. As a positive interaction control, yeast cells were cotransformed with pDHB1‐largeT and pDSL‐p53 vectors. The primers used in the Y2H assays are listed in Table S6.

## Conflict of interest

The authors declare no conflict of interests.

## Supporting information


**Figure S1** Phylogenetic tree based on OsPIL15 homologs in rice and *Arabidopsis*.
**Figure S2** Expression of *OsPIL15* in transgenic rice.
**Figure S3** Comparison of amino acid sequences between the wild‐type (WT) and knockout (KO) lines.
**Figure S4** Phenotype comparisons of transgenic and wild‐type (WT) rice plants.
**Figure S5** Histological analysis of the endosperm.
**Figure S6** Real‐time RT‐PCR validation of the RNA‐Seq results.
**Figure S7** Gene ontology (GO) functional classification of differentially expressed genes (DEGs) in the *OsPIL15*‐OX and *OsPIL15*‐KO lines.
**Figure S8** Identification of the target genes of OsPIL15.
**Figure S9** OsPIL15 affects isopentenyl adenosine (iPA) by regulating expression levels of *OsPUP7*.
**Figure S10** OsPIL15 interacts weakly with OsPGL1, with no interaction with OsPGL2 in yeast cells.

**Table S1** Grain size and 1000‐grain weight in the wild‐type (WT) and transgenic lines.
**Table S2** Plant height and tiller number in the wild‐type (WT) and transgenic lines.
**Table S3** Grain yield of wild‐type (WT) and transgenic lines grown in the field trials.
**Table S4** Expression levels of various genes regulated by *OsPIL15*.
**Table S5** Probes used in the electrophoresis mobility shift assay (EMSA). The motifs are underlined. Mutated nucleotides are shown in red.
**Table S6** List of primers used in this study.
**Table S7** The endogenous gene, synthetic gene and protein sequences of *OsPIL15*.
