# Supplementary material for: The basic helix‐loop‐helix transcription factor, OsPIL15, regulates grain size via directly targeting a purine permease gene OsPUP7 in rice
Source: Plant Biotechnol J. 2019 Jan 24;17(8):1527–37. doi: 10.1111/pbi.13075 (PMC6662305; doi:10.1111/pbi.13075)
Supplement: Supplementary file 1 — Figure S1 Phylogenetic tree based on OsPIL15 homologs in rice and Arabidopsis. Figure S2 Expression of OsPIL15 in transgenic rice. Figure S3 Comparison of amino acid sequences between the wild‐type (WT) and knockout (KO) lines. Figure S4 Phenotype comparisons of transgenic and wild‐type (WT) rice plants. Figure S5 Histological analysis of the endosperm. Figure S6 Real‐time RT‐PCR validation of the RNA‐Seq results. Figure S7 Gene ontology (GO) functional classification of differentially expressed genes (DEGs) in the OsPIL15‐OX and OsPIL15‐KO lines. Figure S8 Identification of the target genes of OsPIL15. Figure S9 OsPIL15 affects isopentenyl adenosine (iPA) by regulating expression levels of OsPUP7. Figure S10 OsPIL15 interacts weakly with OsPGL1, with no interaction with OsPGL2 in yeast cells. [file PBI-17-1527-s002.pdf]

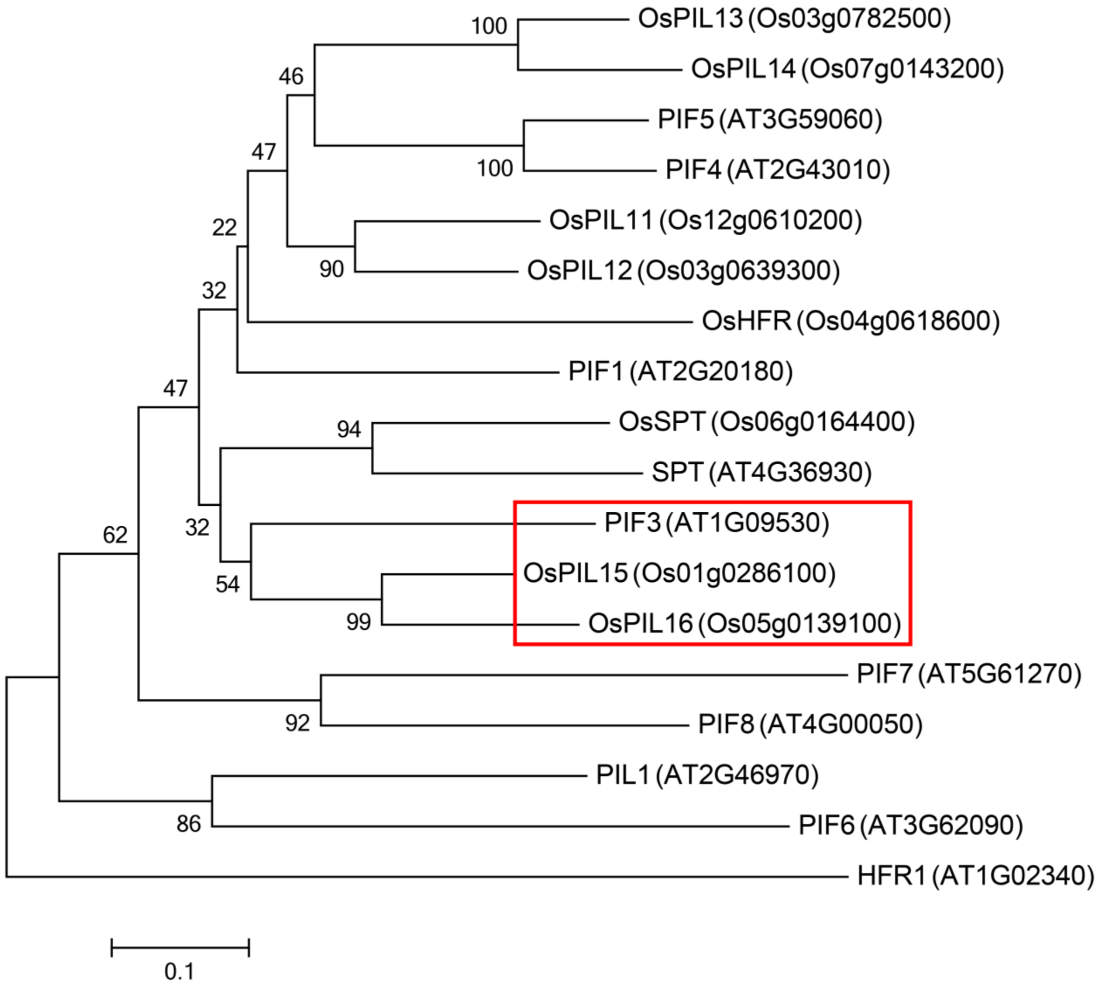


**Figure S1**. Phylogenetic tree based on OsPIL15 homologs in rice and *Arabidopsis*. The phylogenetic tree was constructed using the neighbor-joining method of MEGA6 program. Numbers at nodes indicate the percentage of 1,000 bootstrap replicates. The scale bar at the bottom represents the genetic distance.

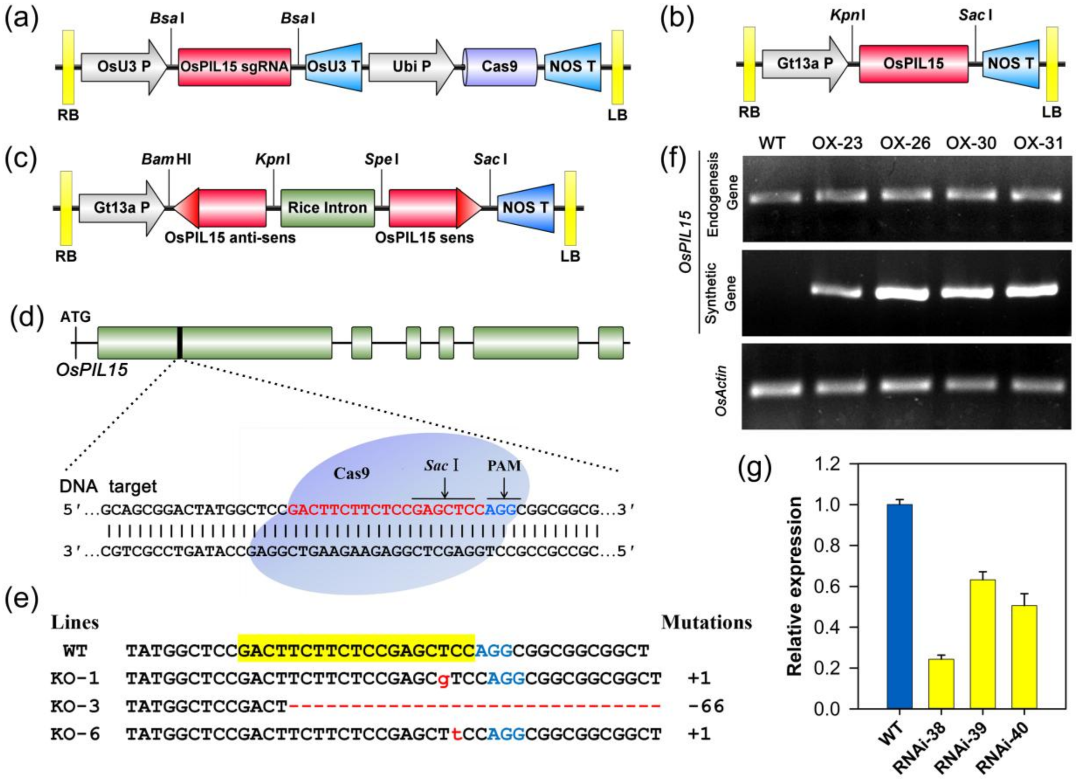


**Figure S2**. Expression of *OsPIL15* in transgenic rice. (a–c) Graphical representation of the construct used to obtain the *OsPIL15*-knock-out (KO), *OsPIL15*-overexpressing (OX), and *OsPIL15*-RNAi lines, respectively. (d) Diagram of the sgRNA:Cas9 complex targeting the rice *OsPIL15* gene. The target site is shown in red and the protospacer-adjacent motif (PAM) is shown in blue. (e) Analysis of mutation sequences in the *OsPIL15*-KO lines. Yellow highlighting denotes sgRNA, red dashes represent deleted bases and insertion nucleotides are shown in red lowercase. (f) Semi-quantitative RT-PCR expression analysis of *OsPIL15* endogenous and synthetic gene expression in the rice endosperm of OX plants compared with wild-type (WT). *OsActin* was used as a loading control. (g) Quantitative RT-PCR analysis of *OsPIL15* endogenous gene expression in the rice endosperm of RNAi plants compared with the WT.

```
                 *        20         *        40         *        60         *        80         *
WT    : MSDGNDFAELLWENGQAVVHGRKKHPQPAFPPFGFFGGTGGGGGGSSSRAQERQPGGIDAFAKVGGGFGALGMAPAVHDFASGFGATTQDNG :  92
KO-1 : MSDGNDFAELLWENGQAVVHGRKKHPQPAFPPFGFFGGTGGGGGGSSSRAQERQPGGIDAFAKVGGGFGALGMAPAVHDFASGFGATTQDNG :  92
KO-3 : MSDGNDFAELLWENGQAVVHGRKKHPQPAFPPFGFFGGTGGGGGGSSSRAQERQPGGIDAFAKVGGGFGALGMAPAVHDFASGFGATTQDNG :  92
KO-6 : MSDGNDFAELLWENGQAVVHGRKKHPQPAFPPFGFFGGTGGGGGGSSSRAQERQPGGIDAFAKVGGGFGALGMAPAVHDFASGFGATTQDNG :  92

             100         *       120         *       140         *       160         *       180
WT    : DDDTVPWIHYPIIDDEDAAAPAALAAADYGSDFFSELQAAAAAAAAAAPPTDLASLPASNHNGATNNRNAPVATTTTREPSKESHGGLSVPT : 184
KO-1 : DDDTVPWIHYPIIDDEDAAAPAALAAADYGSDFFSERPGGGGCRGGRRAADRSRLSASLQSQRRHQ*------------------------- : 158
KO-3 : DDDTVPWIHYPIIDDEDAAAPAALAAADYGSD----------------------SLPASNHNGATNNRNAPVATTTTREPSKESHGGLSVPT : 162
KO-6 : DDDTVPWIHYPIIDDEDAAAPAALAAADYGSDFFSELPGGGGCRGGRRAADRSRLSASLQSQRRHQ*------------------------- : 158

             *       200         *       220         *       240         *       260         *
WT    : TRAEPQPQPQLAAAKLPRSSGSGGGEGVMNFSLFSRPAVLARATLESAQRTQGTDNKASNVTASNRVESTVVQTASGPRSAPAFADQRAAAW : 276
KO-1 : -------------------------------------------------------------------------------------------- :   -
KO-3 : TRAEPQPQPQLAAAKLPRSSGSGGGEGVMNFSLFSRPAVLARATLESAQRTQGTDNKASNVTASNRVESTVVQTASGPRSAPAFADQRAAAW : 254
KO-6 : -------------------------------------------------------------------------------------------- :   -

         280         *       300         *       320         *       340         *       360
WT    : PPQPKEMPFASTAAAPMAPAVNLHHEMGRDRAGRTMPVHKTEARKAPEATVATSSVCSGNGAGSDELWRQQKRKCQAQAECSASQDDDLDDE : 368
KO-1 : -------------------------------------------------------------------------------------------- :   -
KO-3 : PPQPKEMPFASTAAAPMAPAVNLHHEMGRDRAGRTMPVHKTEARKAPEATVATSSVCSGNGAGSDELWRQQKRKCQAQAECSASQDDDLDDE : 346
KO-6 : -------------------------------------------------------------------------------------------- :   -

         *       380         *       400         *       420         *       440         *       460
WT    : PGVLRKSGTRSTKRSRTAEVHNLSERRRRDRINEKMRALQELIPNCNKIDKASMLDEAIEYLKTLQLQVQMMSMGTGLCIPPMLLPTAMQHL : 460
KO-1 : -------------------------------------------------------------------------------------------- :   -
KO-3 : PGVLRKSGTRSTKRSRTAEVHNLSERRRRDRINEKMRALQELIPNCNKIDKASMLDEAIEYLKTLQLQVQMMSMGTGLCIPPMLLPTAMQHL : 438
KO-6 : -------------------------------------------------------------------------------------------- :   -

                 *       480         *       500         *       520         *       540         *
WT    : QIPPMAHFPHLGMGLGYGMGVFDMSNTGALQMPPMPGAHFPCPMIPGASPQGLGIPGTSTMPMFGVPGQTIPSSASSVPPFASLAGLPVRPS : 552
KO-1 : -------------------------------------------------------------------------------------------- :   -
KO-3 : QIPPMAHFPHLGMGLGYGMGVFDMSNTGALQMPPMPGAHFPCPMIPGASPQGLGIPGTSTMPMFGVPGQTIPSSASSVPPFASLAGLPVRPS : 530
KO-6 : -------------------------------------------------------------------------------------------- :   -

             560         *       580         *       600         *       620         *
WT    : GVPQVSGAMANMVQDQQQGIANQQQQCLNKEAIQGANPGDSQMQIIMQGDNENFRIPSSAQTKSSQFSDGTGKGTNARERDGAET* : 637
KO-1 : -------------------------------------------------------------------------------------- :   -
KO-3 : GVPQVSGAMANMVQDQQQGIANQQQQCLNKEAIQGANPGDSQMQIIMQGDNENFRIPSSAQTKSSQFSDGTGKGTNARERDGAET* : 615
KO-6 : -------------------------------------------------------------------------------------- :   -
```

**Figure S3**. Comparison of amino acid sequences between the wild-type (WT) and knock-out (KO) lines. Yellow highlighting represents amino acid deletion or translation termination.

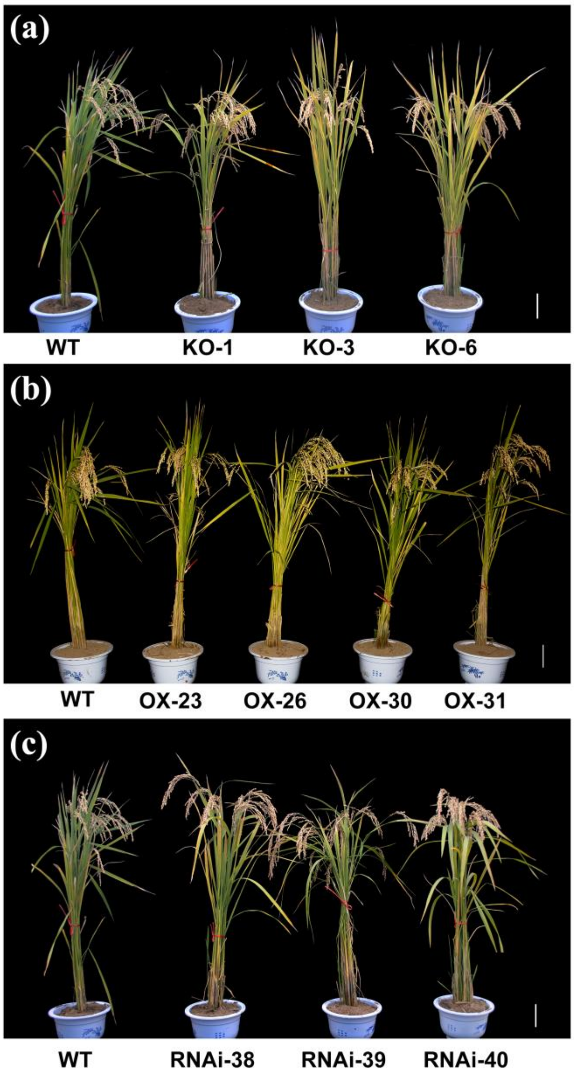


**Figure S4**. Phenotype comparisons of transgenic and wild-type (WT) rice plants. Scale bars: 10 cm. (a) WT and *OsPIL15*-knock-out (KO) lines. (b) WT and *OsPIL15*-overexpressing (OX) lines. (c) WT and *OsPIL15*-RNAi lines.

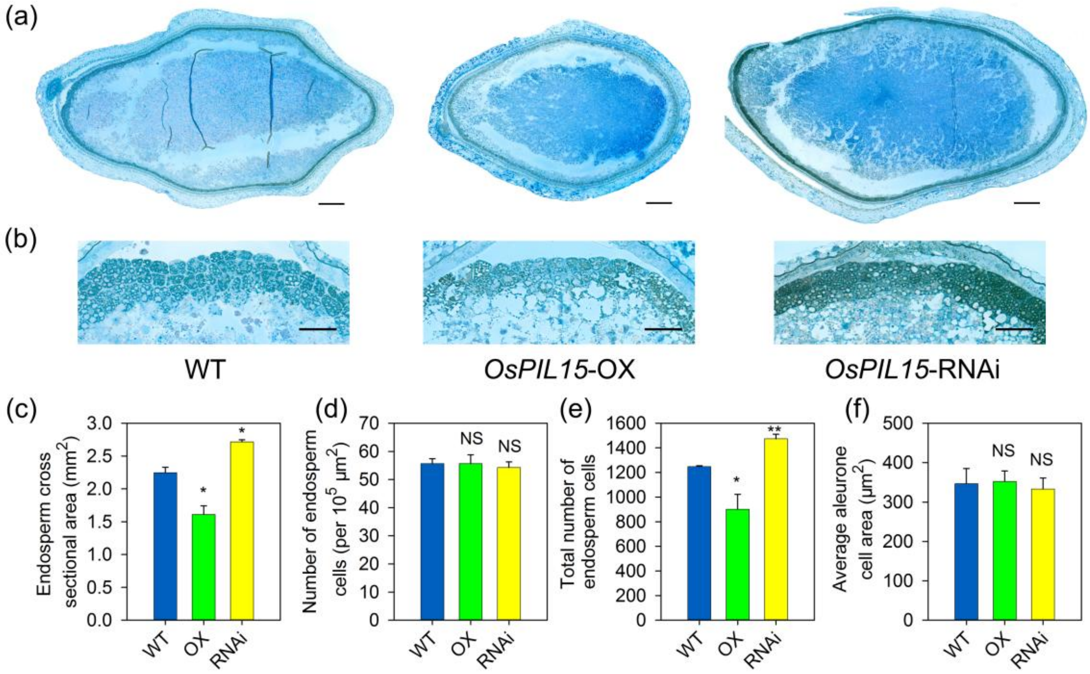


**Figure S5**. Histological analysis of the endosperm. (a) Cross-section of endosperm from wild-type (WT), *OsPIL15*-overexpressing (OX), and *OsPIL15*-RNAi lines. Scale bar: 200 μm. (b) Magnified view of aleurone cells in the dorsal endosperm from WT, *OsPIL15*-OX, and *OsPIL15*-RNAi lines. Scale bar: 50 μm. (c–f) Comparison of endosperm cross sectional areas, number of endosperm cells and average aleurone cell area in WT, *OsPIL15*-OX, and *OsPIL15*-RNAi lines. * $P < 0.05$, ** $P < 0.01$. NS: not significant.

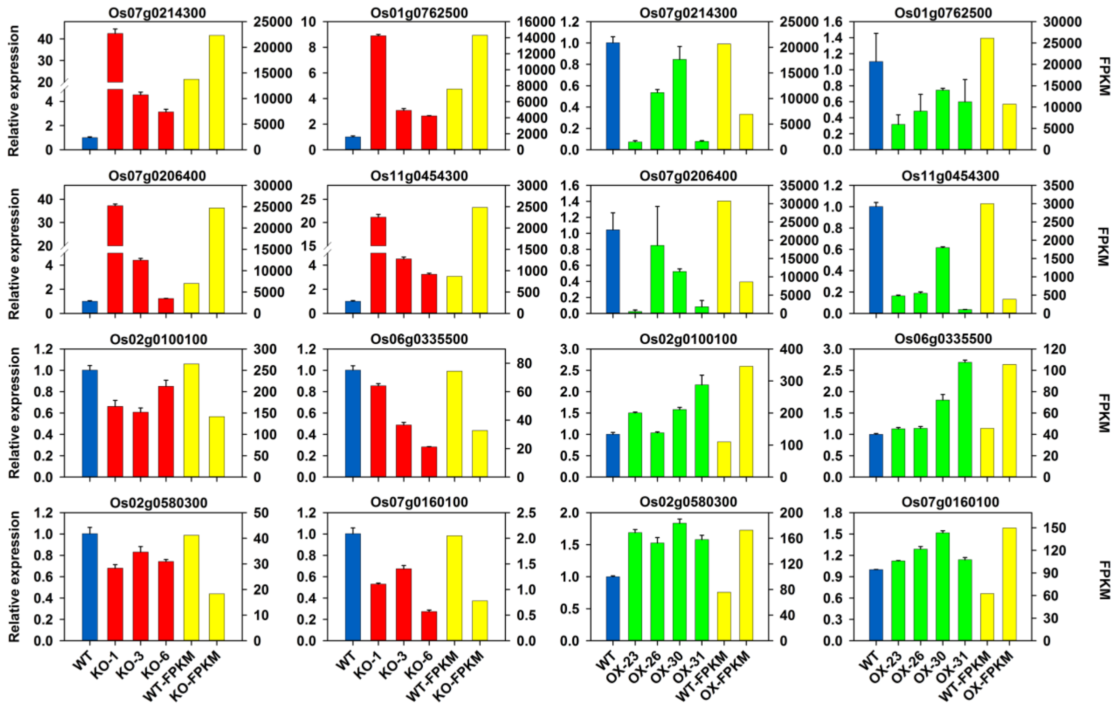


**Figure S6**. Real-time RT-PCR validation of the RNA-Seq results. Fragments per kilobase of exon per million fragments of mapped reads (FPKM) are shown in yellow.

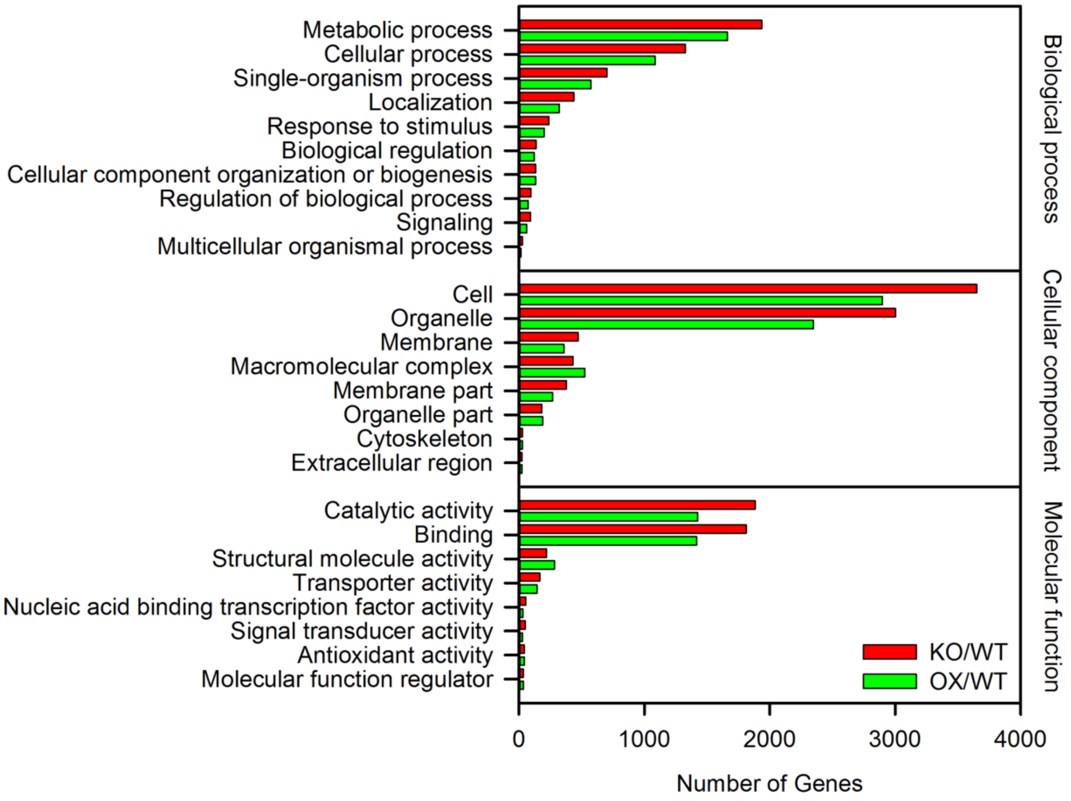


**Figure S7.** Gene ontology (GO) functional classification of differentially expressed genes (DEGs) in the *OsPIL15*-OX and *OsPIL15*-KO lines. The X-axis shows the number of DEGs and the Y-axis represents GO terms.

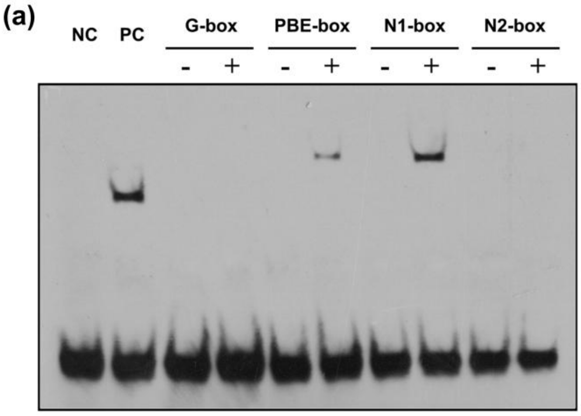


(b)

| Gene | Sequence including N1-box (CACGCG) | Position (from 5′ UTR) |
|---|---|---|
| *OsUGE1*-1 | GAGCACGCACGCGGCAGGAG | -1950 to -1930 |
| *OsUGE1*-2 | CCGTACGCACGCGCCATGCG | -1845 to -1825 |
| *OsXTH9* | AGCAGATCACGCGCGCGGTG | -1175 to -1155 |
| *OsXTH11* | GCGTAGGCACGCGGCGGCTG | -139 to -119 |
| *OsARF3* | CAGGCAGGCACGCGTAGGGG | -909 to -889 |
| *OsARF25* | CGTGGTCCACGCGCGGCATG | -2274 to -2254 |
| *OsPUP7* | AATACAGCACGCGCTATCTC | -2980 to -2970 |
| *OsSAUR22* | TGTTATCCACGCGAAAAGGG | -2387 to -2367 |
| *OsRAB21* | AGTGGTTCACGCGGAGTTCC | -1589 to -1579 |

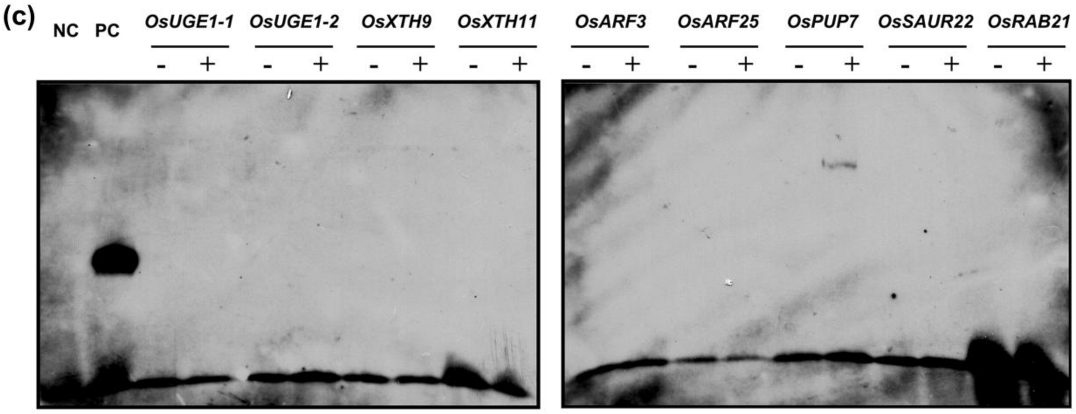


**Figure S8.** Identification of the target genes of OsPIL15. (a) Nucleotide sequences of regions containing a possible G-box, PBE-box, N1-box, or N2-box were used as probes in the electrophoresis mobility shift assay (EMSA). Negative (NC) and positive controls (PC) were used to validate the EMSA system. “-” and “+” denote the absence or presence of OsPIL15 protein. (b) Promoter analysis of the probe sequences including an N1-box. (c) Identification of the target genes of OsPIL15.

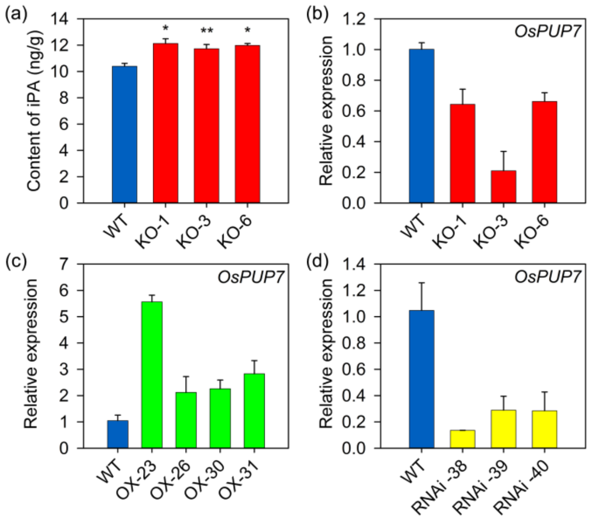


**Figure S9.** OsPIL15 affects isopentenyl adenosine (iPA) by regulating expression levels of *OsPUP7*. (a) Endogenous levels of iPA in the spikelets of wild-type (WT) and *OsPIL15*-KO lines. Data represent means ± SEM (n=3). * $P < 0.05$; ** $P < 0.01$. (b–d) Relative transcription levels of *OsPUP7* in the *OsPIL15*-KO, *OsPIL15*-OX, and *OsPL15*-RNAi lines.

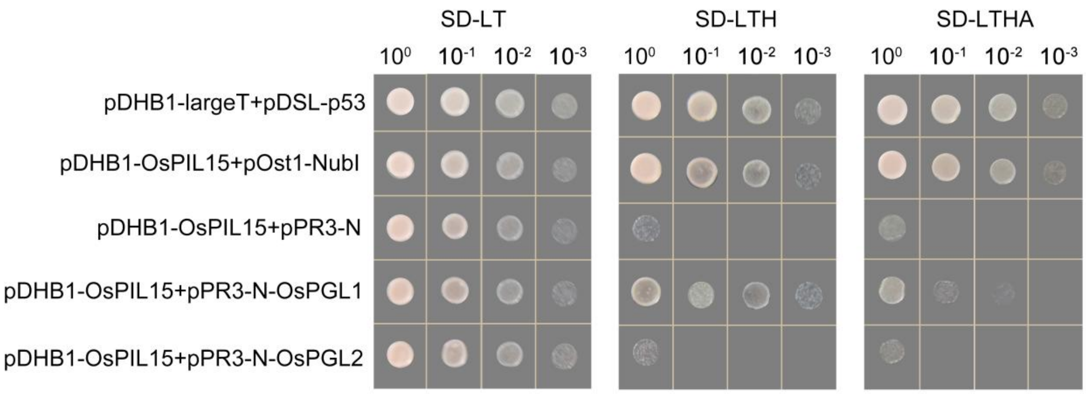


**Figure S10**. OsPIL15 interacts weakly with OsPGL1, with no interaction with OsPGL2 in yeast cells. Interactions between bait and prey were examined in control LT media (SD/-Leu/-Trp), selective LTH media (SD/-Leu/-Trp/-His), and selective LTHA media (SD/-Ade/-His/-Leu/-Trp).
