# Supplementary material for: The basic helix‐loop‐helix transcription factor, OsPIL15, regulates grain size via directly targeting a purine permease gene OsPUP7 in rice
Source: Plant Biotechnol J. 2019 Jan 24;17(8):1527–37. doi: 10.1111/pbi.13075 (PMC6662305; doi:10.1111/pbi.13075)
Supplement: Supplementary file 2 — Table S1 Grain size and 1000‐grain weight in the wild‐type (WT) and transgenic lines. Table S2 Plant height and tiller number in the wild‐type (WT) and transgenic lines. Table S3 Grain yield of wild‐type (WT) and transgenic lines grown in the field trials. Table S4 Expression levels of various genes regulated by OsPIL15. Table S5 Probes used in the electrophoresis mobility shift assay (EMSA). The motifs are underlined. Mutated nucleotides are shown in red. Table S6 List of primers used in this study. Table S7 The endogenous gene, synthetic gene and protein sequences of OsPIL15. [file PBI-17-1527-s001.pdf]

Table S1. Grain size and 1000-grain weight in the wild-type (WT) and transgenic lines.

**Sample**	**Line**	**Grain length (mm)**	**Grain width (mm)**	**1000-grain weight (g)**
Unhusked rice T_1 2016-Year	WT	7.29±0.03	3.29±0.05	23.67±0.57
	KO-1	7.54±0.06*	3.43±0.03*	26.04±0.80*
	KO-3	7.56±0.05*	3.41±0.01**	25.34±0.27*
	KO-6	7.57±0.05*	3.41±0.01**	25.56±0.36*
Brown rice T_2 2015-Year	WT	5.15±0.02	2.76±0.01	22.29±0.16
	OX-23	5.05±0.05*	2.46±0.06**	17.55±1.00**
	OX-26	5.08±0.01**	2.65±0.04*	21.12±0.47*
	OX-30	5.07±0.04*	2.64±0.02**	20.73±0.49*
	OX-31	5.01±0.03**	2.58±0.02**	19.54±0.29**
	RNAi-38	5.35±0.03**	2.80±0.02ns	23.71±0.52*
	RNAi-39	5.35±0.04**	2.76±0.01ns	23.23±0.28*
	RNAi-40	5.27±0.02**	2.87±0.02**	23.75±0.18**
Brown rice T_3 2016-Year	WT	5.21±0.22	2.70±0.01	20.34±0.26
	OX-23	5.05±0.03**	2.39±0.03**	16.04±0.33**
	OX-26	5.06±0.05*	2.52±0.07*	16.89±1.28*
	OX-30	5.02±0.03**	2.63±0.02*	19.74±0.12*
	OX-31	5.11±0.01**	2.52±0.01**	17.25±0.22**
	RNAi-38	5.46±0.05**	2.91±0.02**	25.09±0.69**
	RNAi-39	5.29±0.01*	2.77±0.01*	21.56±0.16*
	RNAi-40	5.43±0.02**	2.96±0.02**	23.96±0.18**

Data represent means ± SEM (n=5). ns: not significant. * $P < 0.05$, ** $P < 0.01$.

Table S2. Plant height and tiller number in the wild-type (WT) and transgenic lines.

Year	Generation	Line	Height (cm)	Tiller number
2016		WT	104.16±1.07	14.57±1.02
	T_1	KO-1	93.65±1.45**	14.50±1.11ns
		KO-3	90.82±1.30**	14.50±2.66ns
		KO-6	95.44±2.75**	15.20±1.20ns
	T_3	OX-23	100.25±1.61ns	15.46±1.20ns
		OX-26	105.51±1.55ns	16.67±0.58ns
		OX-30	104.66±1.72ns	14.82±0.85ns
		OX-31	99.94±2.37ns	16.75±0.82ns
		RNAi-38	102.55±1.51ns	14.40±1.25ns
		RNAi-39	101.80±1.28ns	17.00±1.61ns
		RNAi-40	105.96±1.24ns	13.78±0.98ns
2017		WT	102.27±0.45	12.29±0.58
	T_2	KO-1	94.88±0.54**	13.85±0.78 ns
		KO-3	92.96±0.62**	11.69±0.93 ns
		KO-6	96.58±0.89**	14.00±0.93 ns
	T_4	OX-23	100.73±0.88ns	13.46±0.77 ns
		OX-26	102.04±0.68ns	12.67±0.63 ns
		OX-30	100.54±1.07ns	12.22±1.26 ns
		OX-31	101.21±0.72ns	13.42±0.68 ns
		RNAi-38	101.21±0.50ns	11.43±0.57 ns
		RNAi-39	102.16±0.69ns	12.33±1.12 ns
		RNAi-40	103.61±0.51ns	11.33±0.76 ns

Data represent means ±SEM (n=10). ns: not significant. ** $P < 0.01$.

Table S3. Grain yield of wild-type (WT) and transgenic lines grown in the field trials.

Line	2016		2017		2018	
	**Grain yield per plant (g)**	**Yield increase (%)**	**Grain yield per plant (g)**	**Yield increase (%)**	**Yield per plot (kg)**	**Yield increase (%)**
WT	26.17±0.93	—	25.13±0.49	—	1.42±0.03	—
KO-1	31.36±2.32*	19.75	28.84±0.90*	14.76	1.60±0.06*	13.07
KO-3	29.98±0.96*	14.50	29.87±2.76*	18.89	1.63±0.06*	14.69
KO-6	31.11±1.62*	18.79	28.87±1.32*	14.89	1.65±0.08*	16.59
OX-23	20.61±1.18**	-21.30	22.30±0.88*	-11.25	0.81±0.09**	-43.18
OX-26	21.01±1.98*	-19.78	20.96±1.86*	-16.59	1.12±0.08*	-21.06
OX-30	22.25±0.80**	-15.03	22.68±1.02*	-9.72	1.31±0.01*	-7.77
OX-31	20.50±1.21**	-21.74	21.73±1.25*	-13.54	1.26±0.03*	-11.41
RNAi-38	31.74±2.07*	21.20	33.81±1.59**	34.54	1.73±0.12*	22.08
RNAi-39	30.55±1.06**	16.65	29.09±1.20**	15.78	1.62±0.07^{NS}	14.33
RNAi-40	36.30±2.75**	38.61	32.73±3.00*	30.26	1.66±0.02*	17.35

Values shown are the means ±SEM (n=10 plants, n=3 plots). * $P < 0.05$, ** $P < 0.01$.

Table S4. Expression levels of various genes regulated by *OsPIL15*. Log_2FC (FC, fold changes in the respective gene expression of *OsPIL15*-OX to WT or *OsPIL15*-KO to WT). "-" and "+" denote the absence or presence of N1-box in genes promoter.

Gene name	Locus ID	*OsPIL15*-OX/WT		*OsPIL15*-KO/WT		N1-box in promoter	Description
		Log_2 FC	Up-Down	Log_2 FC	Up-Down		
**Cell development**							
OsKRP6	Os09g0459900	1.42	Up	-1.36	Down	−	Similar to cyclin-dependent kinase inhibitor 6
OsUGE1	Os05g0595100	-2.68	Down	1.31	Up	+	Uridine-diphospho-(UDP)-glucose 4-epimerase
OsCESA4	Os01g0750300	-1.41	Down	0.70	Up	−	Similar to cellulose synthase
OsCSLF6	Os08g0160500	-0.64	Down	1.42	Up	−	Similar to cellulose synthase-like CslF6
OsXTH9	Os04g0604300	-2.17	Down	3.59	Up	+	Similar to xyloglucan endotransglucosylase
OsXTH11	Os06g0696400	-1.27	Down	4.22	Up	+	Xyloglycan endo-transglycosylase precursor
OsCLP	Os01g0937050	-2.75	Down	9.25	Up	+	Homolog of xylanase inhibitor
OsVPE3	Os02g0644000	-1.98	Down	0.63	Up	−	Vacuolar-processing enzyme precursor
**Seed storage proteins**							
OsGluA	Os10g0400200	-1.05	Down	0.84	Up	−	Glutelin type II precursor
OsGluA1	Os01g0762500	-1.29	Down	0.92	Up	−	Glutelin subunit mRNA
OsGluB5	Os02g0268100	-0.73	Down	1.09	Up	+	Similar to glutelin
OsGluC	Os02g0453600	-1.02	Down	0.63	Up	−	Similar to glutelin
OsGluD	Os02g0249000	-1.61	Down	1.19	Up	−	Glutelin, seed strage protein
OsPROLM14	Os05g0330600	-2.06	Down	5.08	Up	−	Similar to prolamin
OsPROLM18	Os05g0332000	-0.69	Down	3.22	Up	−	Similar to prolamin precursor
OsPROLM19	Os07g0219250	-0.93	Down	1.92	Up	−	Prolamin precursor
OsPROLM24	Os06g0507200	-1.85	Down	1.17	Up	−	Prolamin precursor
OsPROLM25	Os07g0206400	-1.83	Down	1.82	Up	−	13 kDa prolamin precursor
OsRAG1	Os07g0214100	-1.86	Down	0.88	Up	−	Seed allergenic protein RA17 precursor

OsRAG2	Os07g0214300	-1.57	Down	0.70	Up	−	Seed allergenic protein RAG2 precursor
OsRAL2	Os07g0213800	-0.94	Down	0.61	Up	−	Similar to Allergenic protein
OsRAL5	Os07g0214600	-1.19	Down	0.73	Up	−	Similar to seed allergenic protein RA17 precursor
**Hormone signal transduction**							
OsYUC11	Os12g0189500	-1.27	Down	0.70	Up	−	Similar to flavin monooxygenase
OsARF1	Os01g0236300	0.76	Up	-1.46	Down	−	Similar to auxin response factor 18
OsARF3	Os01g0753500	1.14	Up	-1.44	Down	+	Auxin response factor 3
OsARF24	Os12g0479400	0.67	Up	-1.06	Down	−	Similar to auxin response factor 24
OsARF25	Os12g0613700	0.97	Up	-1.48	Down	+	Similar to auxin response factor 25
OsIAA1	Os03g0742900	1.46	Up	-1.15	Down	−	Aux /IAA protein
OsIAA8	Os02g0723400	2.75	Up	-1.39	Down	−	Similar to isoform 2 of auxin-responsive protein IAA8
OsIAA21	Os06g0335500	1.21	Up	-1.18	Down	−	Similar to auxin-responsive protein IAA21
OsIAA30	Os12g0601300	3.71	Up	-1.11	Down	−	Similar to auxin-responsive protein (Aux/IAA)
OsSAUR22	Os04g0662200	6.44	Up	-2.98	Down	+	Auxin responsive SAUR protein family protein
OsSAUR37	Os09g0437100	2.48	Up	-0.67	Down	−	Similar to SAUR33-auxin-responsive SAUR family member
OsCKX11	Os08g0460600	1.08	Up	-1.85	Down	−	Similar to cytokinin dehydrogenase 11
OsPUP7	Os05g0556800	2.33	Up	-1.02	Down	+	Purine permease 7
OsAHP1	Os08g0557700	1.24	Up	-0.60	Down	−	Cytokinin signaling and stress response
OsAHP2	Os09g0567400	0.64	Up	-9.00	Down	−	Cytokinin signaling and stress response
OsYABBY1	Os07g0160100	1.26	Up	-1.39	Down	−	Feedback regulation of gibberellin biosynthesis
OsGA2ox10	Os05g0208500	0.72	Up	-0.76	Down	−	Gibberellin 2-beta-dioxygenase 1
OsGID2	Os02g0580300	1.19	Up	-0.66	Down	+	Positive regulator of GA signaling
OsGID1L2	Os09g0461500	2.77	Up	-1.56	Down	−	Alpha/beta hydrolase fold-3 domain containing protein
OsABA8ox1	Os02g0703600	-2.68	Down	4.55	Up	−	Similar to abscisic acid 8'-hydroxylase 1
OsPYL8	Os06g0528300	-0.96	Down	0.65	Up	−	Similar to polyketide cyclase
OsRAB21	Os11g0454300	-2.96	Down	1.52	Up	+	Similar to water-stress inducible protein RAB21

OsWR2	Os06g0604000	2.37	Up	-1.65	Down	+	Similar to ethylene response factor 1
OsWR3	Os02g0797100	1.90	Up	-2.23	Down	−	Rice wax synthesis regulatory gene 3
OsMHZ4	Os01g0128300	1.41	Up	-1.00	Down	+	Similar to predicted protein
OsSub1B	Os09g0286600	1.57	Up	-0.66	Down	−	ERF domain containing protein
OsRTH2	Os05g0539800	1.25	Up	-1.09	Down	−	Reversion-to-ethylene sensitivity1 homologue 2

Table S5. Probes used in the electrophoresis mobility shift assay (EMSA). The motifs are underlined. Mutated nucleotides are shown in red.

Probe	Forward Sequence (5'–3')	Reverse Sequence (5'–3')
G-box	TCTAATGACACGTGAGCAAGTCC	GGACTTGCTCACGTGTCATTAGA
PBE-box	TCTAATGACACATGAGCAAGTCC	GGACTTGCTCATGTGTCATTAGA
N1-box	TCTAATGACACGCGAGCAAGTCC	GGACTTGCTCGCGTGTCATTAGA
N2-box	TCTAATGACACGAGAGCAAGTCC	GGACTTGCTCTCGTGTCATTAGA
OsUGE1-1	GAGCACGCACGCGGCAGGAG	CTCCTGCCGCGTGCGTGCTC
OsUGE1-2	CCGTACGCACGCGCCATGCG	CGCATGGCGCGTGCGTACGG
OsXTH9	AGCAGATCACGCGCGCGGTG	CACCGCGCGCGTGATCTGCT
OsXTH11	GCGTAGGCACGCGGCGGCTG	CAGCCGCCGCGTGCCTACGC
OsARF3	CAGGCAGGCACGCGTAGGGG	CCCCTACGCGTGCCTGCCTG
OsARF25	CGTGGTCCACGCGCGGCATG	CATGCCGCGCGTGGACCACG
OsPUP7	AATACAGCACGCGCTATCTC	GAGATAGCGCGTGCTGTATT
OsSAUR22	TGTTATCCACGCGAAAAGGG	CCCTTTTCGCGTGGATAACA
OsRAB21	AGTGGTTCACGCGGAGTTCC	GGAACTCCGCGTGAACCACT
Mutant probe 1	AATACAGCCGCGACTATCTC	GAGATAGTCGCGGCTGTATT
Mutant probe 2	AATACAGGCAGCCCTATCTC	GAGATAGGGCTGCCTGTATT

Table S6. List of primers used in this study.

Name	Forward primer (5'–3')	Reverse primer (5'–3')
**sgRNA fragment amplification**		
OsPIL15-sgRNA	GGCGGACTTCTTCTCCGAGCTCC	AAACGGAGCTCGGAGAAGAAGTC
**RNAi fragment amplification**		
OsPIL15-RNAi-1	GTACGGTACCCTGGCACAAGCACCATGC	AGCTGGATCCATCTGAAAATTGACTGCTTTTTGTT
OsPIL15-RNAi-2	GTACACTAGTCTGGCACAAGCACCATGC	AGCTGAGCTCATCTGAAAATTGACTGCTTTTTGTT
**Subcellular localization analysis**		
OsPIL15-GFP	CAGTGGTCTCACAACATGAGCGATGGGAACGACTT	CAGTGGTCTCATACATGTCTCGGCCCCATCGCGCT
**Electrophoresis mobility shift assays (EMSA)**		
pET28a-OsPIL15	CAAATGGGTCGCGGATCCATGAGCGATGGGAACGACTTC	GAGTGCGGCCGCAAGCTTTCATGTCTCGGCCCCATCGC
**Yeast one-hybrid assays (Y1H)**		
pGADT7-OsPIL15	ATGGCCATGGAGGCCAGGAATTCATGAGCGATGGGAACGACTTCG	GCAGCTCGAGCTCGATGGATCCTGTCTCGGCCCCATCGCG
**ChIP-qPCR assays**		
OsPUP7-BS	CGCTATCTCTTGTTGTCAGACG	CTAATTGGGATGATAGATGCGTGTTC
**Transient expression assays**		
pGreen II 62-SK-OsPIL15	TCCCCCGGGCTGCAGGAATTCATGAGCGATGGGAACGACT	GTCGACGGTATCGATAAGCTTTCATGTCTCGGCCCCATC
**Yeast two-hybrid assays (Y2H)**		
pDHB1-OsPIL15	TGTCTCCTAAGAACGCGGCCATTACGGCCATGAGCGATGGGAACGACTTCGC	GGGATCCCCCCCGACATGGCCGAGGCGGCCGAGTCTCGGCCCCATCGCGCT
pPR3-N-OsPGL1	GGTATCAACGCAGAGTGGCCATTACGGCCATGGCTAAGTGGGGGTGT	GATATCGAATTCTCGAGAGGCCGAGGCGGCCCTACATCAGAAGGCTGCG
pPR3-N-OsPGL2	GGTATCAACGCAGAGTGGCCATTACGGCCATGTCGAGCAGAAGGTCGT	GATATCGAATTCTCGAGAGGCCGAGGCGGCCTCAGGAGCGGAGGATGCT
**Semi-quantitative RT-PCR and quantitative (q) RT-PCR**		
Endogenous *OsPIL15*	TGGCTCATTTCCCTCATCTC	ATTCGCTATGCCTTGTTGCT
Synthetic *OsPIL15*	TGCCAGTCCACAAGACAGAG	AGCTGCAGGGTTTTCAGGTA
Fragment *OsPIL15*	CCAACATGGTGCAAGATCAG	ATTGGCTGCTTTTTGTCTGG
OsRAG2 (Os07g0214300)	GAGGCATCTACAGGGAGCTC	AGTTCTCGGGGTCCTAGGAT
OsGluA1 (Os01g0762500)	GGGAGAGGTATAACAGGGCC	GCACCAATGAGCTACACCAG
OsPROLM25 (Os07g0206400)	GCGCGGTTTGATCCTCTTAG	CTGCTGCATGACTTGGTTGT
OsRAB21 (Os11g0454300)	GGAGGAGGAAGAAGGGGATC	CATGATGCCCTTCTTCTCGC

OsPDIL1-4 (Os02g0100100)	GAAAGCATCTCCGTGGCATT	TACATCTCCACCACCGTACG
OsIAA21 (Os06g0335500)	GAAGGCACAGGTGGTAGGAT	ACCATCCATGCTCACCTTGA
OsGID2 (Os02g0580300)	AGAGAGCCGTGGTAATGAGG	AAGACCTTGGACTCTGGAGC
OsYABBY1 (Os07g0160100)	TGCTCTCCATGGATCTTGCT	TAGGTGACACTTGCTGCTGA
OsPUP7 (Os05g0556800)	GCCTGCTGTCGAGGTTCTAC	GTACGCGAAGAGCAGGTTGT
OsActin	GGAAGTACAGTGTCTGGATTGGAG	TCTTGGCTTAGCATTCTTGGGT

Table S7. The endogenous gene, synthetic gene, and protein sequences of *OsPIL15*.

Sequences

Endogenous gene sequence

ATGTCCGACGGCAACGACTTCGCCGAGCTGCTGTGGGAGAACGGCCAGGCGGTGGTGCACGGGAGG
AAGAAGCACCCGCAGCCGGCCTTCCCGCCGTTCGGCTTCTTCGGTGGCACCGGCGGTGGCGGCGGCG
GCAGCAGTAGTAGAGCCCAGGAGAGGCAGCCCGGCGGCATCGATGCGTTCGCCAAGGTGGGGGGCG
GCTTCGGCGCCTTGGGCATGGCTCCGGCGGTGCACGACTTCGCTTCTGGCTTCGGCGCCACCACGCAG
GACAACGGTGATGATGACACCGTTCCGTGGATCCATTACCCCATAATTGACGATGAAGACGCCGCCG
CCCCTGCTGCTCTCGCAGCAGCGGACTATGGCTCCGACTTCTTCTCCGAGCTCCAGGCGGCGGCGGCT
GCCGCGGCGGCCGCCGCGCCGCCGACCGATCTCGCCTCTCTGCCAGCCTCCAATCACAACGGCGCCA
CCAATAACAGAAATGCTCCGGTTGCCACCACCACCACCAGGGAACCCTCCAAGGAAAGCCACGGCG
GCCTGTCGGTTCCCACCACCCGAGCCGAGCCGCAGCCGCAGCCACAGCTCGCCGCAGCCAAGCTGCC
TCGGTCGAGCGGCAGCGGCGGCGGCGAGGGCGTGATGAACTTCTCGCTCTTCTCCCGCCCGGCCGTC
CTGGCGAGGGCGACGCTGGAGAGCGCGCAGAGGACGCAGGGCACCGACAATAAGGCGTCCAATGTC
ACCGCGAGCAACCGCGTCGAGTCGACGGTCGTGCAGACGGCGAGCGGGCCAAGGAGCGCACCGGCG
TTCGCCGATCAGAGGGCGGCGGCGTGGCCGCCGCAGCCGAAGGAGATGCCGTTCGCGTCCACGGCA
GCCGCTCCCATGGCCCCGGCCGTTAACCTGCACCACGAGATGGGCCGTGACAGGGCAGGCCGAACCA
TGCCTGTCCACAAAACCGAGGCGAGGAAGGCACCTGAGGCCACGGTCGCGACATCGTCGGTGTGCTC
CGGCAACGGAGCTGGGAGTGACGAGCTGTGGCGCCAGCAGAAGCGGAAGTGCCAGGCCCAGGCAGA
GTGCTCAGCTAGCCAAGACGATGATCTTGACGATGAACCTGGAGTATTGAGAAAATCTGGAACCAGG
AGCACGAAACGCAGCCGCACAGCTGAGGTTCACAATTTATCAGAAAGGAGGAGAAGGGACAGGATC
AATGAAAAGATGCGCGCTCTGCAAGAACTCATTCCCAACTGCAACAAGATTGATAAAGCCTCGATGC
TGGATGAAGCTATAGAGTACCTCAAAACCCTTCAGCTTCAAGTACAGATGATGTCCATGGGAACTGG
GCTGTGCATTCCTCCAATGCTATTACCAACAGCCATGCAGCACTTGCAAATTCCACCGATGGCTCATT
TCCCTCATCTCGGCATGGGATTGGGGTACGGGATGGGCGTCTTCGACATGAGCAACACTGGAGCACT
TCAGATGCCACCCATGCCTGGTGCTCACTTTCCCTGCCCAATGATCCCAGGTGCGTCACCACAAGGTC
TTGGGATCCCTGGCACAAGCACCATGCCAATGTTTGGGGTTCCTGGGCAAACAATTCCTTCGTCAGCG
TCTAGTGTACCACCATTTGCATCTTTGGCTGGTCTTCCTGTTAGGCCAAGCGGGGTCCCTCAAGTATC
AGGCGCCATGGCTAACATGGTGCAAGACCAGCAACAAGGCATAGCGAATCAACAGCAGCAATGTCT
GAACAAGGAAGCTATACAGGGAGCAAATCCAGGTGATTCACAAATGCAGATCATCATGCAGGGTGA
CAACGAGAATTTTAGGATACCCTCTTCAGCCCAAACAAAAAGCAGTCAATTTTCAGATGGTACCGGC
AAGGGGACCAACGCTAGAGAGAGAGATGGGGCTGAAACATAA

Synthetic gene sequence

ATGAGCGATGGGAACGACTTCGCGGAACTCCTGTGGGAGAATGGGCAAGCGGTCGTCCACGGCCGC
AAGAAACATCCGCAGCCGGCCTTCCCGCCATTTGGCTTCTTTGGCGGCACAGGGGGCGGCGGCGGGG
GCAGCTCCAGCAGGGCCCAAGAAAGGCAGCCAGGGGGCATCGATGCGTTTGCGAAAGTGGGCGGCG
GCTTTGGGGCGCTCGGCATGGCGCCGGCGGTCCATGATTTTGCGTCCGGCTTCGGCGCCACCACCCA
AGATAACGGCGATGATGATACCGTCCCGTGGATCCACTATCCGATCATCGACGACGAAGATGCCGCC
GCGCCAGCGGCCCTCGCGGCCGCGGACTACGGGTCCGATTTTTTCTCCGAACTGCAAGCCGCCGCGG
CGGCCGCGGCCGCGGCCGCCCCACCAACAGATCTCGCCTCCCTCCCAGCCTCCAACCACAACGGCGC
GACCAACAACAGGAACGCCCCAGTCGCCACCACAACCACCAGGGAGCCATCCAAGGAGAGCCATGG
GGGGCTCTCCGTGCCAACCACAAGGGCCGAGCCACAGCCACAACCACAGCTGGCGGCCGCCAAACT
CCCAAGGAGCAGCGGGAGCGGCGGCGGCGAGGGGGTCATGAATTTCTCCCTCTTTTCCCGCCCAGCC

GTCCTCGCCAGGGCCACACTGGAATCCGCGCAACGCACACAGGGGACAGACAATAAGGCCAGCAAC
GTCACCGCGTCCAATCGCGTGGAGAGCACCGTGGTCCAAACAGCGAGCGGCCCAAGGTCCGCCCCA
GCCTTTGCGGATCAGCGCGCCGCGGCCTGGCCACCACAGCCAAAGGAAATGCCGTTTGCCAGCACAG
CGGCGGCCCCAATGGCGCCAGCCGTCAATCTCCACCATGAGATGGGCCGCGATAGGGCGGGCAGGA
CAATGCCAGTCCACAAGACAGAGGCCCGCAAAGCCCCAGAAGCGACCGTCGCCACAAGCTCCGTGT
GTAGCGGGAATGGCGCCGGGAGCGACGAACTGTGGAGGCAACAGAAGAGGAAGTGTCAAGCGCAG
GCCGAATGCAGCGCGTCCCAAGACGACGACCTCGATGATGAGCCAGGGGTCCTCCGCAAAAGCGGC
ACACGCTCCACAAAGAGGAGCCGCACCGCCGAGGTCCATAACCTCTCCGAGCGCAGGAGGCGCGAT
CGCATCAACGAGAAAATGCGCGCGCTCCAAGAGCTGATCCCAAACTGCAACAAGATCGACAAGGCC
AGCATGCTCGACGAGGCCATCGAGTACCTGAAAACCCTGCAGCTCCAGGTGCAGATGATGAGCATGG
GCACAGGGCTCTGCATCCCACCGATGCTGCTGCCGACAGCCATGCAACACCTGCAGATCCCACCGAT
GGCCCATTTCCCACATCTCGGCATGGGGCTGGGGTATGGGATGGGGGTGTTCGACATGTCCAACACC
GGCGCGCTGCAAATGCCGCCAATGCCAGGGGCCCACTTTCCGTGCCCAATGATTCCAGGCGCCTCCC
CACAGGGCCTCGGCATTCCAGGCACAAGCACAATGCCGATGTTCGGCGTGCCGGGGCAAACCATTCC
AAGCAGCGCCTCCAGCGTCCCACCATTTGCCAGCCTCGCCGGGCTCCCAGTGAGGCCATCCGGCGTC
CCACAAGTCAGCGGGGCGATGGCCAACATGGTGCAAGATCAGCAGCAGGGGATTGCCAACCAGCAG
CAACAATGCCTGAACAAGGAAGCGATCCAAGGGGCGAACCCAGGGGACAGCCAGATGCAGATCATC
ATGCAGGGCGACAACGAGAACTTTCGCATCCCGAGCAGCGCCCAGACAAAAAGCAGCCAATTCAGC
GATGGCACCGGCAAGGGGACAAATGCCAGGGAGCGCGATGGGGCCGAGACATGA

Protein sequence

MSDGNDFAELLWENGQAVVHGRKKHPQPAFPPFGFFGGTGGGGGGSSSRAQERQPGGIDAFAKVGGGF
GALGMAPAVHDFASGFGATTQDNGDDDTVPWIHYPIIDDEDAAAPAALAAADYGSDFFSELQAAAAAA
AAAAPPTDLASLPASNHNGATNNRNAPVATTTTREPSKESHGGLSVPTTRAEPQPQPQLAAAKLPRSSGS
GGGEGVMNFSLFSRPAVLARATLESAQRTQGTDNKASNVTASNRVESTVVQTASGPRSAPAFADQRAAA
WPPQPKEMPFASTAAAPMAPAVNLHHEMGRDRAGRTMPVHKTEARKAPEATVATSSVCSGNGAGSDEL
WRQQKRKCQAQAECSASQDDDLDDEPGVLRKSGTRSTKRSRTAEVHNLSERRRRDRINEKMRALQELIP
NCNKIDKASMLDEAIEYLKTLQLQVQMMSMGTGLCIPPMLLPTAMQHLQIPPMAHFPHLGMGLGYGMG
VFDMSNTGALQMPPMPGAHFPCPMIPGASPQGLGIPGTSTMPMFGVPGQTIPSSASSVPPFASLAGLPVRP
SGVPQVSGAMANMVQDQQQGIANQQQQCLNKEAIQGANPGDSQMQIIMQGDNENFRIPSSAQTKSSQFS
DGTGKGTNARERDGAET
